# Chronic Rhinosinusitis with Nasal Polyps: A Survey on Routine Management and Evaluation of Disease Control in Practice

**DOI:** 10.3390/jpm13111531

**Published:** 2023-10-26

**Authors:** Eugenio De Corso, Giancarlo Ottaviano, Carlotta Pipolo, Elena Cantone, Davide Mattavelli, Matteo Alicandri-Ciufelli, Daniela Lucidi, Marco Caminati, Gianenrico Senna, Carlo Cavaliere, Angelo Ghidini, Stefania Gallo, Alberto Macchi, Sara Torretta, Veronica Seccia, Massimiliano Garzaro, Gian Luca Fadda, Matteo Trimarchi, Ernesto Pasquini, Fabio Pagella, Frank Rikki Canevari, Andrea Preti, Ignazio La Mantia, Jacopo Galli

**Affiliations:** 1 Otolaryngology, Head and Neck Surgery, Rhinology, A. Gemelli University Hospital Foundation, IRCSS, 00168 Rome, Italy; eugenio.decorso@gmail.com; 2 Department of Neurosciences, Otolaryngology Section, University of Padova, 35122 Padova, Italy; 3 Otolaryngology Unit, ASST Santi Paolo e Carlo, Department of Health Sciences, University of Milan, 20142 Milan, Italy; 4 Department of Neuroscience, Reproductive and Odontostomatological Sciences-ENT Section, University of Naples 29 Federico II, 80131 Naples, Italy; 5 Unit of Otorhinolaryngology-Head and Neck Surgery, ASST Spedali Civili Brescia, Department of Medical and Surgical Specialties, Radiological Sciences, and Public Health, University of Brescia, 25123 Brescia, Italy; 6 Department of Otorhinolaryngology Head and Neck Surgery, Azienda Ospedaliero-Universitaria Policlinico di Modena, 41124 Modena, Italy; 7 Department of Medicine, University of Verona and Verona University Hospital, 37134 Verona, Italy; 8 Department of Sense Organs, Sapienza University of Rome, 00185 Rome, Italy; 9 ENT Department, Azienda USL Reggio Emilia-IRCCS, 42123 Reggio Emilia, Italy; 10 Otorinolaryngology Unit, Head and Neck Department, ASST Sette Laghi and UPLOAD Research Center, University of Insubria, 21100 Varese, Italy; stefania.gallo@asst-settelaghi.it (S.G.); macchi.doc@gmail.com (A.M.); 11 Fondazione IRCCS Ca’ Granda Ospedale Maggiore Policlinico, Department of Clinical Sciences and Community Health, University of Milan, 20122 Milan, Italy; 12 Otolaryngology, Audiology and Phoniatric Operative Unit, Department of Surgical, Medical, Molecular Pathology and Critical Care Medicine, Azienda Ospedaliera Universitaria Pisana, University of Pisa, 56124 Pisa, Italy; v.seccia@ao-pisa.toscana.it; 13 Department of Otorhinolaryngology, Rhinology Unit, Eastern Piedmont University-Maggiore Hospital, 28100 Novara, Italy; 14 Department of Otorhinolaryngology, San Luigi Gonzaga Hospital, University of Turin, 10043 Orbassano, Italy; g.fadda@sanluigi.piemonte.it; 15 Otorhinolaryngology Unit, Head and Neck Department, IRCCS San Raffaele Scientific Institute, Vita-Salute San Raffaele University, 20132 Milan, Italy; 16 Otorhinolaryngology Unit, Dip Chirurgie Specialistiche, Ospedale Bellaria AUSL BO, 47814 Bologna, Italy; 17 Department of Clinical, Surgical, Diagnostic and Pediatric Sciences, University of Pavia, 27100 Pavia, Italy; 18 Otorhinolaryngology Unit, IRCCS Policlinico San Martino Genova, Dipartimento 1 DISC Università di Genova, 16124 Gernova, Italy; c.rikki@libero.it; 19 Department of Otorhinolaryngology, Ospedale San Giuseppe IRCCS Multimedica Milan, 20099 Milan, Italy; 20 Department of Medical-Surgical Sciences and Advanced Technologies-“G.F. Ingrassia” Director of E.N.T. Unit P.O. “G. Rodolico” University of Catania, 95123 Catania, Italy; ilamantia@unict.it; 21 Department of Head, Neck and Sensory Organs, Catholic University of The Sacred Heart, 00168 Rome, Italy

**Keywords:** CRSwNP, disease control, disease severity, biologics, endoscopic sinus surgery

## Abstract

Chronic rhinosinusitis with nasal polyps (CRSwNP) is a disease with a significant impact on quality of life. The overall goal of CRSwNP management, as with other chronic conditions, is to achieve “disease control”, and for that reason, a definition of control of disease is pivotal in deciding the best treatment strategy. Although many staging systems have already been developed to evaluate the disease, disease control is not yet to be standardized, and a specific tool that is consistently applied and accepted by all practitioners is still missing in daily clinical practice. To gain an overview of the implementation and limitations of existing guidelines and to shed light on real-life definitions of control and disease severity, we conducted a nationwide survey of otorhinolaryngologists routinely treating CRSwNP to identify unmet clinical needs in Italy. The results showed homogeneous responses regarding the knowledge contained in international guidelines while highlighting the difficulty of their implementation in day-to-day practice. Respondents called attention to the importance of clinical symptoms, giving more weight to the patient’s perspective. Among the symptoms to be considered, respondents emphasized nasal obstruction, followed by loss of sense of smell and rhinorrhea. Others also believe that the physician’s perspective should be considered, and the inclusion of endoscopy as a measure of control was warranted by many. The need for a specific tool that is able to unequivocally ascertain disease control is increasingly pivotal in this new era of biologics for treating CRSwNP.

## 1. Introduction

Chronic rhinosinusitis with nasal polyps (CRSwNP) affects around 4% of the global population, with a significant impact on the quality of life (QoL) [1,2,3,4,5]. CRSwNP is associated with a substantial burden for patients and healthcare systems, and this burden is further increased in patients with poorly controlled disease [2,3,6,7,8].

The management of CRSwNP has typically consisted of challenging diagnostic workup and medical therapy aiming at suppressing inflammation [1]. For instance, both the 2012 European Position Paper on Rhinosinusitis (EPOS 2012) and EPOS 2020 recommend the use of intranasal corticosteroids (INCSs) and adding oral steroids, depending on the severity of the disease [9,10]. However, the overall goal of CRSwNP management, as well as that for other chronic conditions that cannot be cured, is to achieve “disease control” [3,4]. The definition of “disease control” is therefore critical and likely crucial to set the goal of therapy, and it could be generally defined as the state of the disease in which clinical manifestations have a limited and acceptable impact on patients’ QoL [11].

From a practical point of view, although many staging systems have already been developed for the evaluation of the disease, disease control has not yet been standardized, and a specific tool is still missing in daily clinical practice [3]. Moreover, not only physicians but also patients must be aware of the “concept” of disease control to provide valuable information for self-assessment [12]. Accordingly, the lack of disease control indicates the need to change the treatment, whereas disease control suggests that the therapy can be maintained or possibly de-escalated [12].

However, despite advances in therapy, real-world data have suggested that a large proportion of patients with CRSwNP suffer from uncontrolled disease, which is associated with a significant disease burden requiring long-term follow-up, medication, and frequently repeated sinus surgeries [13]. Disease control should thus drive treatment, especially considering that up to 40% of patients may have uncontrolled disease [14]. Unfortunately, CRSwNP remains a challenge for clinicians to manage adequately, given the difficulty in controlling symptoms long term, especially in the subgroup of patients whose condition is driven by a type 2 inflammatory response [15]. Thus, the personalized management of the individual patient is needed to limit unnecessary and ineffective treatments [15]. This is particularly relevant considering the large variety of treatment options and guidance. The assessment of disease control in CRSwNP is highly variable in clinical practice and often depends on the personal judgment of the clinician [16,17], further complicated by the availability of biologics targeting type 2 inflammation. Biologics now offer new possibilities to achieve adequate disease control even in the most difficult cases, even if their exact position in the treatment algorithm remains somewhat unclear. A rational approach for managing uncontrolled severe CRSwNP has been the subject of much discussion [18]. Recent multidisciplinary consensus and expert opinion have been proposed in this regard [15,18]. However there remains a need for greater consensus in regard to the assessment and management of the disease [12]. There is currently no agreement in the literature on how to assess disease control in CRSwNP. In fact, despite the efforts to define disease control by the EPOS 2012 and 2020 steering group, this definition has not been applied consistently in research, and we imagine also in clinical practice.

For this reason, we believe it is essential to understand current routine management practices. To shed light on clinical practice, with a particular focus on aspects related to the control of CRSwNP, we carried out a nationwide survey of otorhinolaryngologists with the aim of identifying clinically unmet needs. Among the areas queried, we considered the application of guidelines, the definition and assessment of disease control, the definition of disease severity, response to prior therapies, and the treatment of comorbidities.

## 2. Materials and Methods

This was a national survey regarding disease control in CRSwNP and the management of uncontrolled disease. It was drafted and distributed to understand more about daily practice in Italy, with the possibility of identifying unmet clinical needs. The survey was developed by a group of otorhinolaryngologists who are experienced in managing severe CRSwNP; they reviewed the literature and discussed the aspects that should be included in the questionnaire during a meeting in Rome on 7–8 October 2022. The authors were divided into groups and proposed questions for specific survey topics. We focused on 5 main areas: (1) application of guidelines, (2) definition and assessment of disease control, (3) definition of disease severity, (4) response to prior therapies, and (5) treatment of comorbidities. Next, a selection process with critical appraisal of all the questions took place (E.D.C., O.G., C.P., and E.C.), and the final questionnaire was built. Remote approval by all authors produced a final total of 46 questions in Italian that were formatted for style and answer possibilities to ensure direct and standardized responses that reflected the respondents’ experience. The full questionnaire and the complete responses are available in the Supplementary Materials.

The survey was set up on Survey Monkey^®^, and physicians authorized to prescribe biologics for severe uncontrolled CRSwNP and identified in each region from throughout Italy were invited by email to participate, without significant discrepancy in number between the north, center, and south. Item-total reliability and internal consistency reliability were assessed using Cronbach’s alpha coefficient analysis. The Cronbach’s alpha coefficient reached 0.71, indicating satisfactory internal consistency. The survey distribution started on 1 November 2022 and was closed on 31 March 2023. All answers were considered appropriate and were included in the analysis. We also collected data on the characteristics of participants (the first 7 questions). We performed a descriptive analysis and presented some of the most significant results as histograms in the Supplementary Materials. The authors of this manuscript discussed the survey results, and all approved of the final version of the article and the translation of the questionnaires in English.

## 3. Results

A total of 117 participants from 91 national centers of rhinology filled out the survey. The characteristics of the responders and centers are outlined in Table 1. More than half of the participants who practiced in university hospitals (60%) had more than 5 years of experience (50.43%) and were aged under 40 years (58%). These data seem to connote a high interest of young ear, nose, and throat specialists (ENTs) in the topic. The geographic distribution represented the whole country well. The majority (76%) routinely carried out endoscopic nasal surgery. Of note, most ENTs surveyed as authorized to prescribe biologics worked in specialized rhinology outpatient clinics (79.5%), although structured multidisciplinary collaboration existed in just over half (56%).

### 3.1. Application of Guidelines

Most participants declared that they routinely carry out specific assessments for disease control in CRSwNP patients (Q8; 76%). Almost all ENTs agreed or partially agreed that the EPOS 2020 guidelines provided information to control the disease in patients with CRSwNP (Q8; 96.58%). Most clinicians apply the evaluation of control suggested by EPOS guidelines in practice (87%); however, only more than half (Q11; 53%) apply these in all patients with CRSwNP. A large group also considers the EUFOREA indications (64%), although just over one-third (Q12; 37%) reported that they routinely apply EUFOREA guidelines to define uncontrolled disease in all patients. Moreover, when questioned on the shortcomings of the guidelines, participants reported that the guidelines did not contain well-defined characteristics of the uncontrolled patient (43%), differed in terms of cutoff to define uncontrolled disease (52.99%), are poorly applicable to real life (23.9%), and had some inconsistency in their references (11%).

### 3.2. Assessment of Disease Control in CRSwNP in Practice

Most of the survey participants reported that, in assessing disease control, the first criterion to be considered should be the control of symptoms (82%). However, a significant group of participants believe that assessing endoscopic signs of disease (70%) and recovery of olfactory function are also pivotal (Q15). Nasal obstruction was considered the most important symptom (Q16; 94%), followed by impaired sense of smell (80%) and rhinorrhea (65%). The majority (Q18; 72%) also agreed that nasal obstruction, smell, and rhinorrhea can define uncontrolled patients. Finally, the participants agreed that it could be useful to have well-defined scores and cutoff values for each evaluated symptom (Q17; 68%).

Agreement was also reached regarding the consideration that QoL features heavily impact disease control. Sleep, fatigue, work productivity, and alterations of smell were held to be the most important QoL features (Q19).

Regarding the tools to be used and related cutoffs to define severe and uncontrolled disease, participants reported that the Nasal Polyps Score (NPS) (84.62%) and Sino-Nasal Outcome Test on 22 items (SNOT-22) (83; 62%) were the most important (Q21). Considering the cutoff value for the NPS (Q22), most said that a value of 4 or 5 should be considered (63%). For the SNOT-22, most cited a cutoff of 40 or 50 (Q23; 79%). Almost all agreed or partially agreed that, when assessing QoL with SNOT-22, nonspecific sinus domains should be interpreted as closely related to disease and not other causes (Q24). Participants suggested considering a cutoff value > 2 if NCS is used (Q25; 80%). Considering visual analog scale (VAS)’s total symptoms score in defining a severe uncontrolled form of the disease, a wide variety of responses were seen. However, almost all cited >5, 6, or 7 (Q26). For the assessment of olfaction, Sniffin’ Sticks were thought to be the most useful (Q27; 78%), followed by olfactory VAS (30%). Another third of participants considered that inexpensive and rapid semi-objective tests are needed to assess olfaction.

Considering aspects that can influence the assessment of non-control in CRSwNP (Q14), 81% answered that corticosteroid treatment close to or concurrent to the evaluation, along with other treatments, such as recent surgery (54%) and treatment for other comorbidities (60%) needed to be considered as aspects that can influence the assessment of non-control in CRSwNP (Q14).

### 3.3. Evaluation of Previous Surgery in Practice

Almost half of the respondents estimated an uncontrolled disease after surgery in 10–30% of patients (Q29). When assessing patients after surgical intervention (Q28), most felt that the number (77%) and type (74%) of interventions were the most important factors to consider; however, the time after the last intervention was also important (62%). The adequate time interval to evaluate surgical results to define if surgery could not provide good control of disease was 6 or 12 months (Q30; 60%).

Most participants (71%) suggested that a patient must have had adequate surgical intervention to be defined as uncontrolled (Q31). Regarding the definition of adequate intervention, the majority considered a complete functional endoscopic sinus surgery with or without preservation of the middle turbinate adequate (Q32). The majority (Q33; 80%) also believed that a patient can be considered uncontrolled after one adequate intervention. After surgical intervention, the NPS score to define a patient as uncontrolled was mostly >4 (41%) or >5 (28%).

### 3.4. Evaluation of Previous Medical Treatments and Steroids

The definition of uncontrolled disease based on the use of oral corticosteroids (OCSs) in naive patients (Q35) was heterogeneous: the most common criteria were the number of annual cycles (77%) and the total dose in one year (50%). Regarding INCS (Q36), both assessing patients’ treatment compliance and the treatment duration longer than 2 months were widely acknowledged as relevant topics. Most patients could be considered uncontrolled if the patient had undergone at least two previous cycles of an OCS in the last year (Q37; 56%). The vast majority (Q38; 83%) also agreed that complications related to the excessive use of corticosteroids should be routinely investigated in practice. Almost half reported that 5–15 days of OCS therapy should be considered as one course of therapy. However, in accordance with the heterogeneity in prescription timing and dosage of steroids among ENTs in CRSwNP patients, a variety of responses were seen (Q39).

Regarding response to prior biological therapy, 64% reported that an evaluation of associated comorbidities should be included in the definition of disease control (Q40). All participants agreed (85.5%) or partially agreed (14.5%) that having a specific tool for evaluating disease control in clinical practice in CRSwNP patients would be useful (Q41).

### 3.5. Treatment of Comorbidities

The vast majority reported that asthma should be taken into consideration as the most important comorbidity when evaluating patients with severe uncontrolled CRSwNP (Q42; 93%); however, allergies (52%) were also recognized. Almost all agreed that, in patients with asthma as a coexisting disease, asthma control also needs to be defined and assessed (Q43). Similarly, it was widely agreed that assessment by a pulmonary specialist is important to assess asthma control (Q44; 91%). There was also broad agreement that assessment of control in patients with CRSwNP and asthma should be multidisciplinary (Q45) and that a therapeutic strategy that controls both asthma and CRSwNP is important (Q46).

## 4. Discussion

This survey had the main aim of understanding current practice in Italy in defining uncontrolled disease in CRSwNP. Firstly, the characteristics of the participants would indicate that the cohort is well represented in terms of clinical experience, although the average age of the participants suggests a lot of interest in this topic among the younger categories. In addition, the participants were distributed throughout Italy, predominantly in university and public hospitals, suggesting that the responses collected herein reflect routine clinical practice in Italy. Furthermore, the estimates provided by participants regarding the proportion of patients with uncontrolled disease are largely in line with published data [13]. Since up to a third of patients may have uncontrolled disease, this underscores a currently unmet clinical need, although there is little data on this aspect in the literature.

The data gathered in this survey on the use of guidelines underline their importance and that colleagues throughout the country consult them. Nevertheless, although ENT specialists agreed that the European guidelines [3,18] provide useful clinical information on disease control, recommendations are not routinely applied in practice [11]. Indeed, from the survey results, we can argue that, although almost all participants apply the EPOS guidelines in practice (87%), only just more than half (Q11; 53%) apply them to all patients with CRSwNP. Furthermore, some shortcomings of the current guidelines were noted, including the lack of well-defined characteristics for uncontrolled patients, discrepancies among different cutoff scores, and inconsistency in the references. From what has been described, it is clear that experts will soon have to make a further effort to define disease control in CRSwNP, thus increasing the consensus on the definition of uncontrolled severe disease.

In line with the definition of cardinal symptoms of CRSwNP by EPOS 2020 in patients with uncontrolled disease [10], there was also broad agreement that nasal obstruction, olfactory disorders and, lastly, rhinorrhea are the most important symptoms to take into consideration when evaluating disease control in CRSwNP. The participants also agreed that the assessment of QoL and evaluation of endoscopic findings should also be included in the evaluation of disease control, especially if the patient is under treatment with biologics. The results of this survey also show that there is an increasing emphasis in practice on the appraisal of olfactory function, recognizing the important role of its evaluation for the assessment of disease control.

Some heterogeneity in daily practice exists regarding the use of assessment tools. In many centers, olfactory assessment is carried out exclusively by VAS, while the physicians believe that smell should also be evaluated semi-objectively, and Sniffin’ Sticks were thought to be the best means to evaluate olfactory disorders [19,20,21]. This could be related to the limited availability of such tools and the increased evaluation time if using them.

Regarding tools used in practice for assessing the success of treatment with biologics, SNOT-22 and NPS were the most useful [17]. In fact, while VAS symptom scores are still commonly used in daily practice, the NPS and SNOT-22 are most frequently used to assess clinically meaningful changes at established points (e.g., at 6 and 12 months after the start of treatment) to evaluate treatment success [22]. Including these two parameters in the baseline evaluation to assess the potential of treating candidates with severe uncontrolled CRSwNP with biologics is of no less importance. Indeed, this may be one reason explaining the fast uptake of these tools in clinical practice [15,18]. In this regard, some confusion may result from variation in the optimal cutoff values indicated by the position papers [3,23] to define severe uncontrolled CRSwNP. Data from our survey indicate that the most considered cutoffs are >4 or >5 for NPS and >40 or >50 for SNOT-22 [24].

Surgery has a central role in managing uncontrolled CRSwNP. However, despite the postoperative use of topical steroids, polyps still recur in about 40% of patients within 18 months [25]. Surgery is recommended in the current guidelines [3] and has been a standard procedure in the journey of CRSwNP patients, indicating the historic lack of alternative medical treatment options [26]. Indeed, among the participants in this survey, the vast majority declared that they routinely perform endoscopic nasal surgery. To date, there is no broadly accepted definition of an optimal outcome after endoscopic nasal surgery.

Recently, a group proposed that surgical success should be based on subjective data and nasal endoscopy, with subjective and clinically relevant improvement of a validated patient-reported outcome questionnaire, along with a good endoscopic result or a complete subjective resolution if a suboptimal endoscopy result is seen [27]. In regard to considering evaluation after surgical intervention for uncontrolled CRSwNP, most held that the number and types of interventions were the most important parameters to be considered and that the patient must have received at least one surgery to be able to be defined as uncontrolled. Some authors recently suggested [28] that the evaluation of the adequateness of the performed surgery also needs to be included. There are, in fact, several aspects that should be considered: on the one hand, the type of surgery performed; and, on the other hand, the completeness of the surgery itself. Unfortunately, there is currently no unanimous definition of what appropriate surgery means, although some evidence suggests that extent of previous surgery may influence the effectiveness of some biologics.

Another important chapter that needs addressing is the evaluation of steroid use in the patient’s clinical history. Firstly, adherence to INCS was considered to be important by participants in this survey, and they believed that at least 2 months of therapy were needed before considering a patient uncontrolled and eligible for biologics [11]. Interestingly, the evaluation of adverse effects of INCS was less important. This aligns with a previous survey revealing that most prescribers believe that INCSs are not associated with significant systemic adverse events [16]. In contrast, there was also near full agreement that the assessment of complications of therapy with OCS should be performed routinely, including hyperglycemia, gastric reflux, insomnia, hypertension, diabetes, etc. [29]. This shows that there is good knowledge of the potential adverse effects of oral steroids and the consequent need to monitor for adverse events. However, we must not forget that systemic steroids also have their place in disease management, particularly to manage re-exacerbations and relapse using brief cycles [11]. Although half of the participants considered a cycle to be from 5 to 15 days, some disagreed, with some considering longer or shorter durations to constitute a cycle. This stresses that, in daily practice, it is likely that, to a large extent, prescribers rely on their personal clinical experience to manage patients in terms of the number and duration of cycles. However, it must be highlighted that there is no specific guidance on the use of oral steroids in terms of the dose and length of a cycle. Indeed, in daily practice, a previous survey highlighted that prescribing practices were very heterogeneous [16]. In that survey, almost half prescribed 25 mg/day prednisone independently of body weight, and around 30% considered body weight when prescribing; the majority prescribed an oral steroid, ranging from 5 to 15 days. In addition, in that survey, most participants further reported that international recommendations on the use of oral steroids for CRSwNP are not exhaustive [16]. The need for further guidance in this area could be useful in practice.

CRSwNP and asthma are frequently coexisting conditions, and their relationship is receiving increasing attention, especially related to type 2 inflammation [30]. The common and comorbid nature of asthma in patients with CRSwNP was recognized by almost all participants, indicating good knowledge of the disease and its comorbidities. In addition, the need for a therapy that treats both conditions was well recognized. In this regard, the need for multidisciplinary management was recognized by almost all participants, in agreement with recent consensus on the management of patients with CRSwNP [15]. The major limitation of this study is the inclusion of only Italian rhinologists and therefore the analysis of peculiarities of the Italian health care system and approach. However, their needs can surely be indicative and of a great stimulus for the collective rhinologists worldwide.

## 5. Conclusions

It is clear from this survey that there is a broad knowledge of guidelines for uncontrolled CRSwNP and its management. For most areas, this was reflected in the generally high consistency of the responses, which indicates a high degree of homogeneity in routine clinical practice. Based on the Italian experience, a paper was recently published [17] with some practical recommendations on the treatment of the disease, especially considering new therapeutic options, such as biologics. In contrast, with specific regard to the assessment of disease control, the results of this survey and our previous experience show that the definitions in the guidelines are largely known but not applied in clinical practice. This could be related to the lack of a clear definition of disease control in CRSwNP. According to most respondents (82%), assessment should predominantly focus on clinical symptoms, giving more weight to the patient’s perspective. Among the symptoms to be considered, respondents gave importance to nasal obstruction, followed by loss of sense of smell and rhinorrhea. Others also believe that the physician’s perspective should be considered, and thus the endoscopy result should also be included. In addition, this survey shows a lack of a specific tool in clinical practice that clinicians could use more widely.

## Figures and Tables

**Table 1:** Characteristics of participants (n = 117).

	N (%)
Age (years)	
20–30	33 (28.21)
30–40	35 (29.91)
40–50	24 (20.51)
50–60	8 (6.84)
60–70	17 (14.53)
Sex	
Male	69 (58.97)
Female	48 (41.03
Years of experience	
<5	58 (49.57)
5–10	15 (12.82)
10–20	19 (16.24)
>20	25 (21.37)
Type of practice	
Public hospital	38 (32.48
University Hospital	71 (60.68)
Regional clinic	2 (1.71)
Independent Practice	6 (5.13)
Geographic area	
Northeast	15 (12.82)
Northwest	33 (28.21)
Center	39 (33.33)
South	30 (25.64)
Endoscopic sinonasal surgery	
Routinely perform	89 (76.07)
Have never performed	16 (13.68)
Performed in the past but not currently	12 (10.26)
Which best describes your center	
Rhinology outpatient clinic	93 (79.49)
Multidisciplinary collaboration network with allergists and pulmonologists	66 (56.41)

## Data Availability

Not applicable.

## Supplementary Materials

The following supporting information can be downloaded at https://www.mdpi.com/article/10.3390/jpm13111531/s1.
