# Supplementary material for: Chronic Rhinosinusitis with Nasal Polyps: A Survey on Routine Management and Evaluation of Disease Control in Practice
Source: J Pers Med. 2023 Oct 26;13(11):1531. doi: 10.3390/jpm13111531 (PMC10672031; doi:10.3390/jpm13111531)
Supplement: Supplementary file 1 [file jpm-13-01531-s001.zip › jpm-2652716-supplementary.pdf]

D1 Specialist's Age

Responses: 117 Skipped: 0

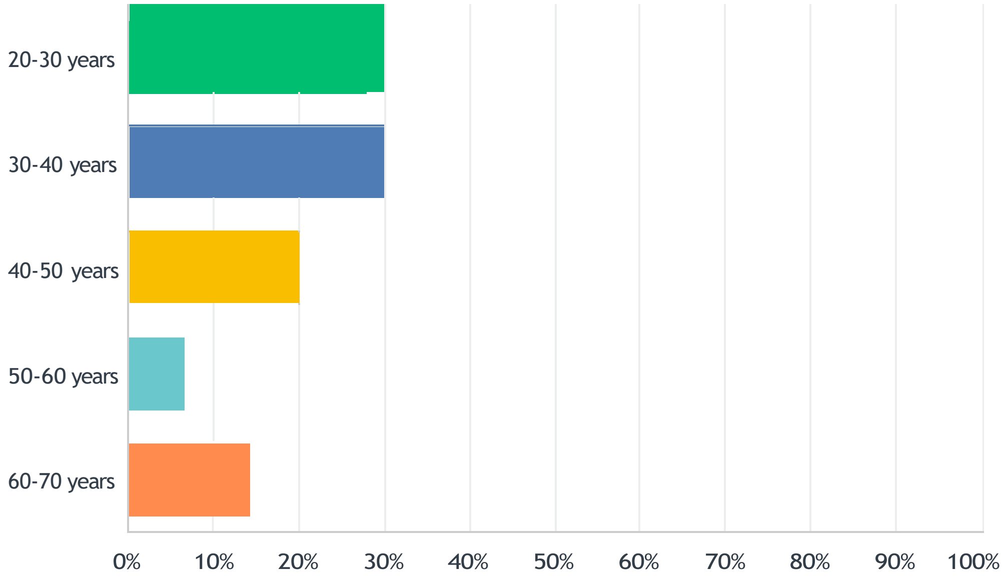

RESPONSE OPTIONS	RESPONSES	
20-30 years	28.21%	33
30-40 years	29.91%	35
40-50 years	20.51%	24
50-60 years	6.84%	8
60-70 years	14.53%	17
TOTAL RESPONDERS		117

D2 Gender

Responses: 117 Skipped: 0

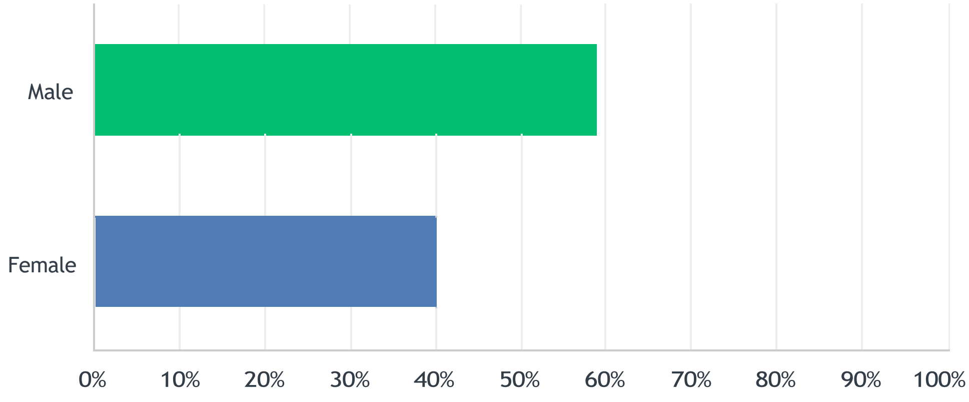

RESPONSE OPTIONS	RESPONSES	
Male	58.97%	69
Female	41.03%	48
TOTAL RESPONDERS		117

D3 I'm ...

Responses: 117   Skipped: 0

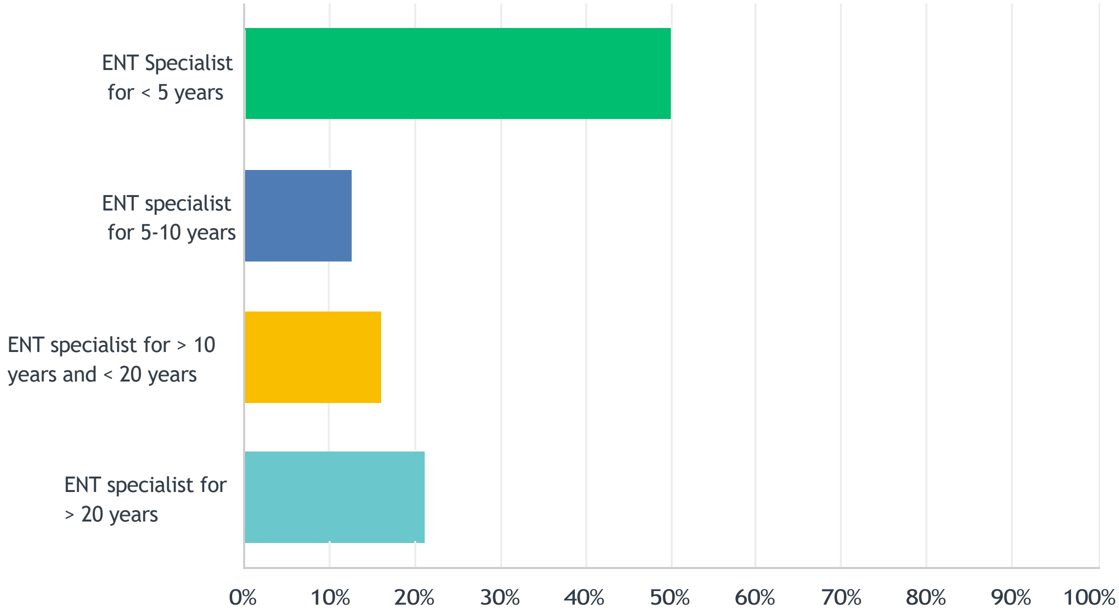

RESPONSE OPTIONS	RESPONSES	
ENT specialist for less than 5 years	49.57%	58
ENT specialist for 5 -10 years	12.82%	15
ENT specialist for more than 10 years and less than 20 years	16.24%	19
ENT specialist for more than 20 years	21.37%	25
TOTAL RESPONDERS		117

D4 I'm ENT specialist and I work at a:

Responses: 117 Skipped: 0

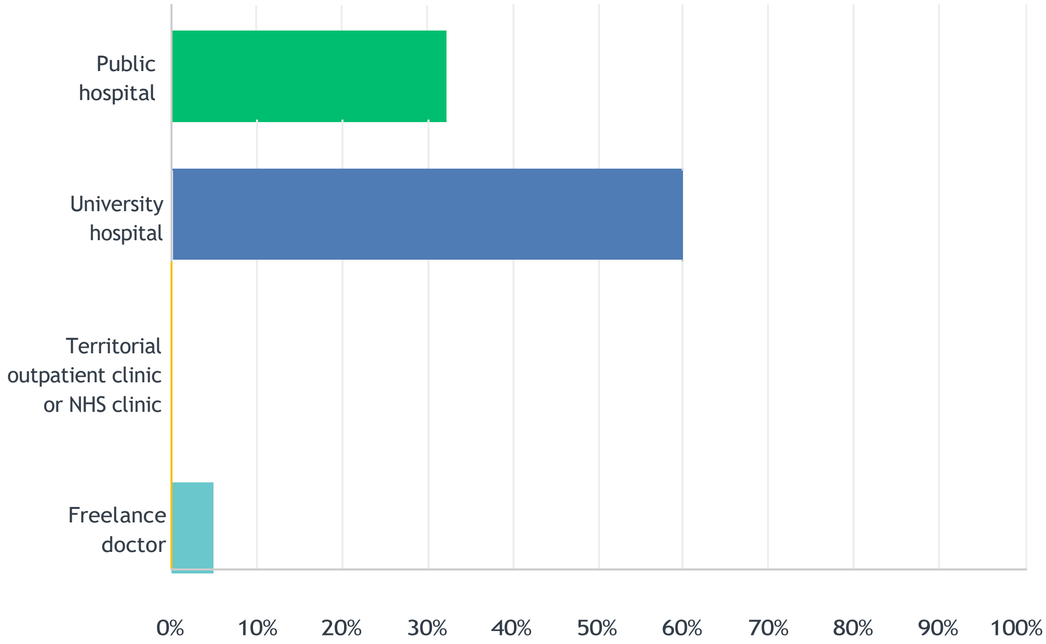

RESPONSE OPTIONS	RESPONSES	
Public hospital	32.48%	38
University Hospital	60.68%	71
Territorial outpatient clinic or NHS outpatient clinic	1.71%	2
Freelance Doctor	5.13%	6
TOTAL RESPONDERS		117

D5 The facility where I work is located in:

Responses: 117 Skipped: 0

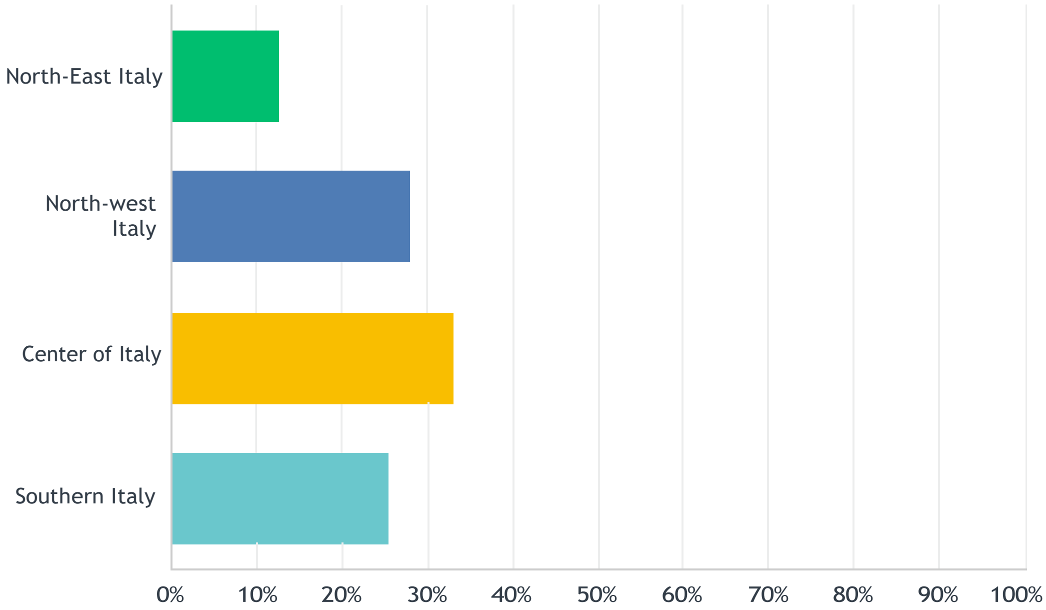

RESPONSE OPTIONS	RESPONSES	
North-east Italy	12.82%	15
North-west Italy	28.21%	33
Center of Italy	33.33%	39
Southern Italy	25.64%	30
TOTAL RESPONDERS		117

D6 Regarding endoscopic sinus surgery:

Responses: 117 Skipped: 0

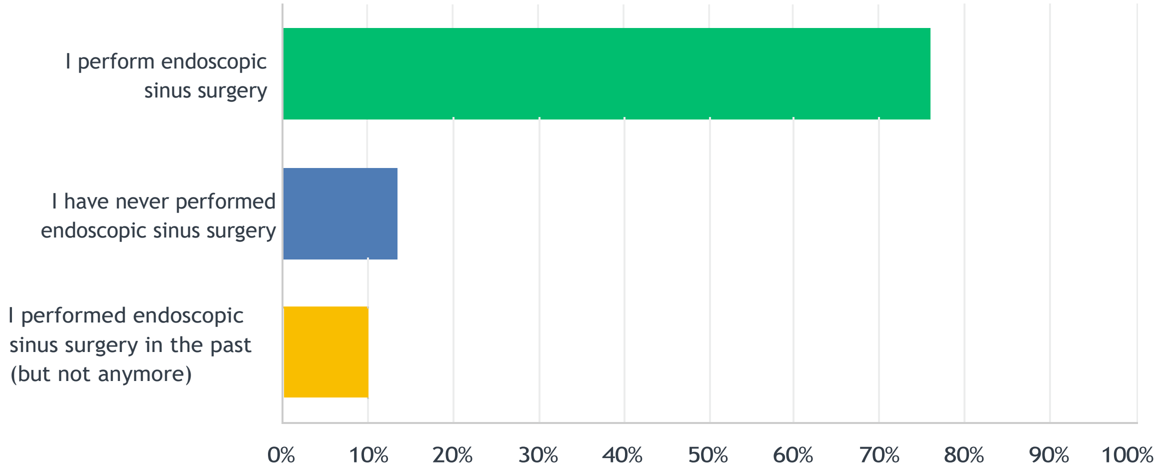

RESPONSE OPTIONS	RESPONSES	
I perform endoscopic naso-sinus surgery	76.07%	89
I have never performed endoscopic naso-sinus surgery	13.68%	16
I performed endoscopic naso-sinus surgery in the past (but not anymore)	10.26%	12
TOTAL RESPONDERS		117

D7 Which of the following better describe your center? (Multiple answers possible)

Responses: 117 Skipped: 0

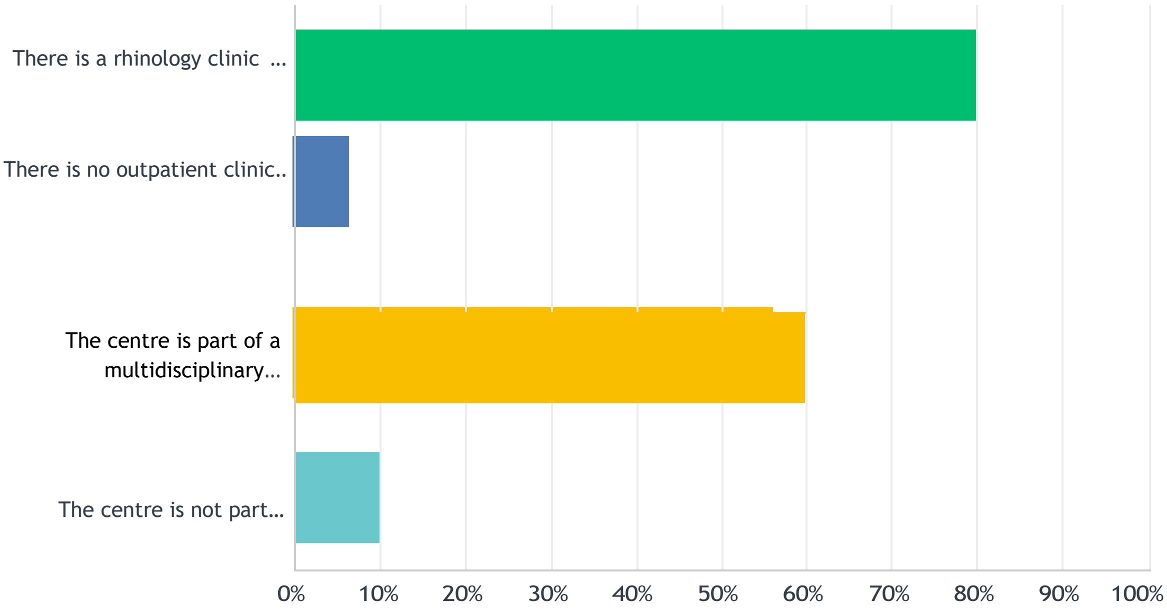

RESPONSE OPTIONS	RESPONSES	
There is a rhinology clinic where patients with chronic recalcitrant rhinosinusitis are treated	79.49%	93
There is no outpatient clinic where patients with chronic recalcitrant rhinosinusitis are treated	6.84%	8
The center is part of a multidisciplinary collaboration network with allergists and pulmonologists	56.41%	66
The center is not part of a multidisciplinary collaboration network	9.40%	11
TOTAL RESPONDERS		117

D8 In your clinical practice do you carry out specific assessment for disease control in chronic rhinosinusitis? (Multiple answers possible)

Responses: 117 Skipped: 0

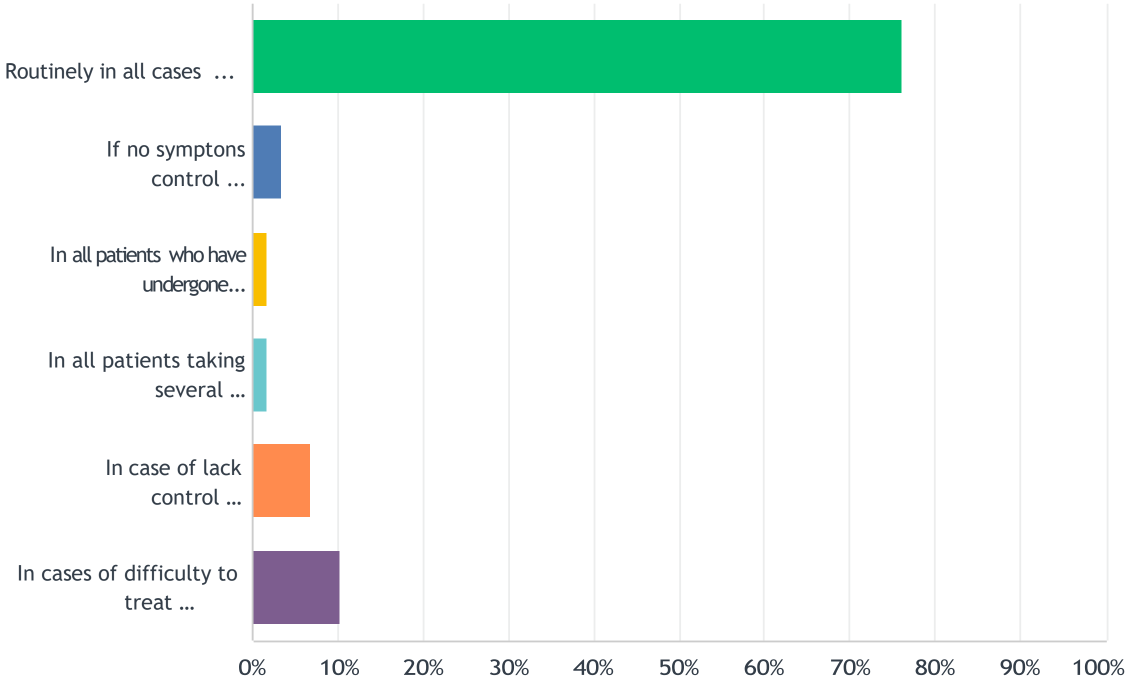

RESPONSE OPTIONS	RESPONSES	
Routinely in all cases of polyposis	76.07%	89
If no sympton control after surgery	3.42%	4
In all patients who have undergone multiple surgeries	1.71%	2
In all patients taking several courses of systemic corticosteroids	1.71%	2
In cases of difficult to treat CRSwNP in candidates for biological therapy	6.84%	8
	10.26%	12
TOTAL RESPONDERS		117

D9 In your routine clinical practice, what is the percentage of patients with severe CRSwNP who are not controlled by systemic corticosteroids?

Responses: 117 Skipped: 0

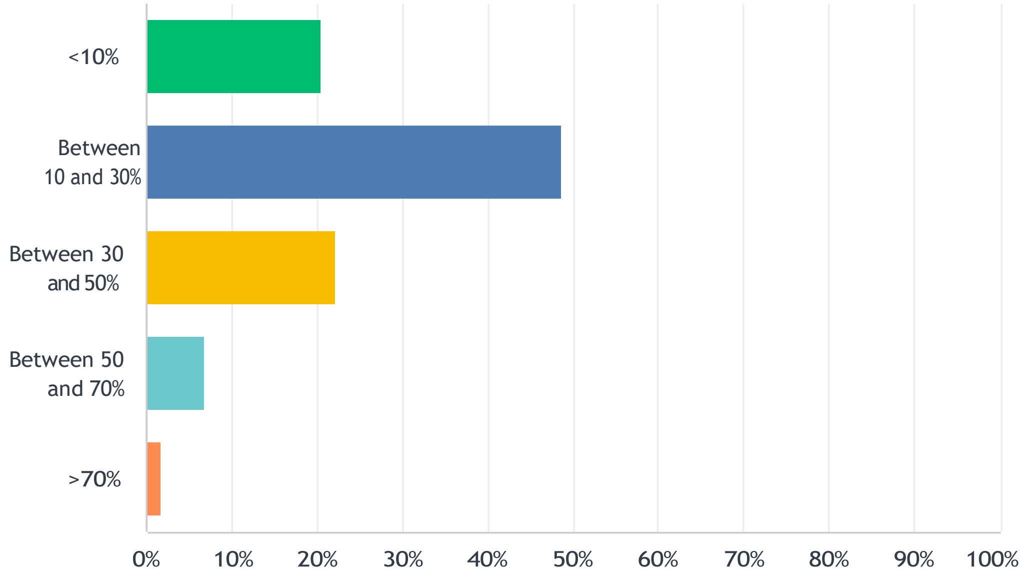

RESPONSE OPTIONS	RESPONSES	
<10%	20.51%	24
Between 10 and 30%	48.72%	57
Between 30 and 50%	22.22%	26
Between 50 and 70%	6.84%	8
>70%	1.71%	2
TOTAL RESPONDERS		117

D10 Do you believe that national and international guidelines provide useful clinical indications to define disease control in CRSwNP in clinical practice?

Responses: 117 Skipped: 0

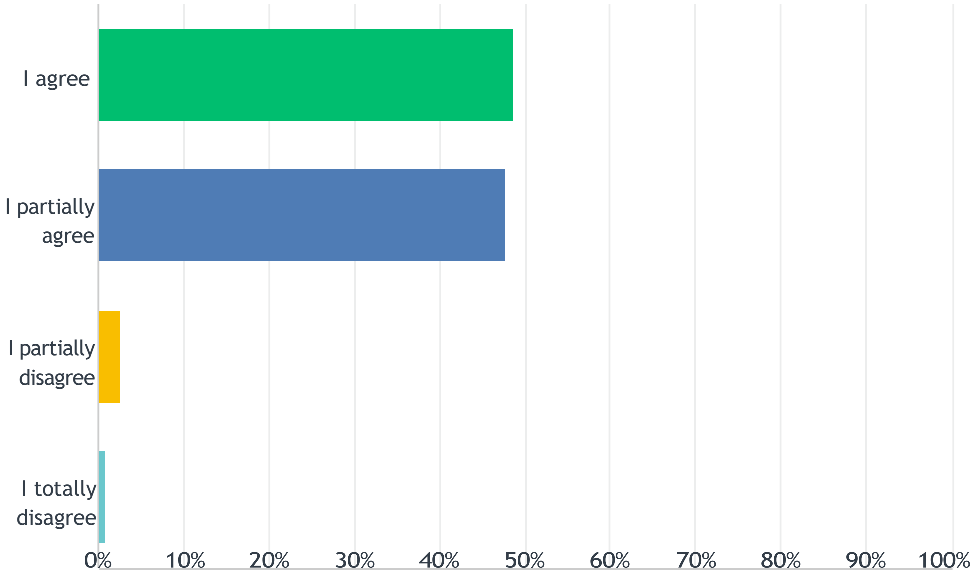

RESPONSE OPTIONS	RESPONSES	
I agree	48.72%	57
I partially agree	47.86%	56
I partially disagree	2.56%	3
I totally disagree	0.85%	1
TOTAL RESPONDERS		117

D11 Do you apply the indications in the EPOS 2020 guidelines in clinical practice to evaluate disease control?

Responses: 117 Skipped: 0

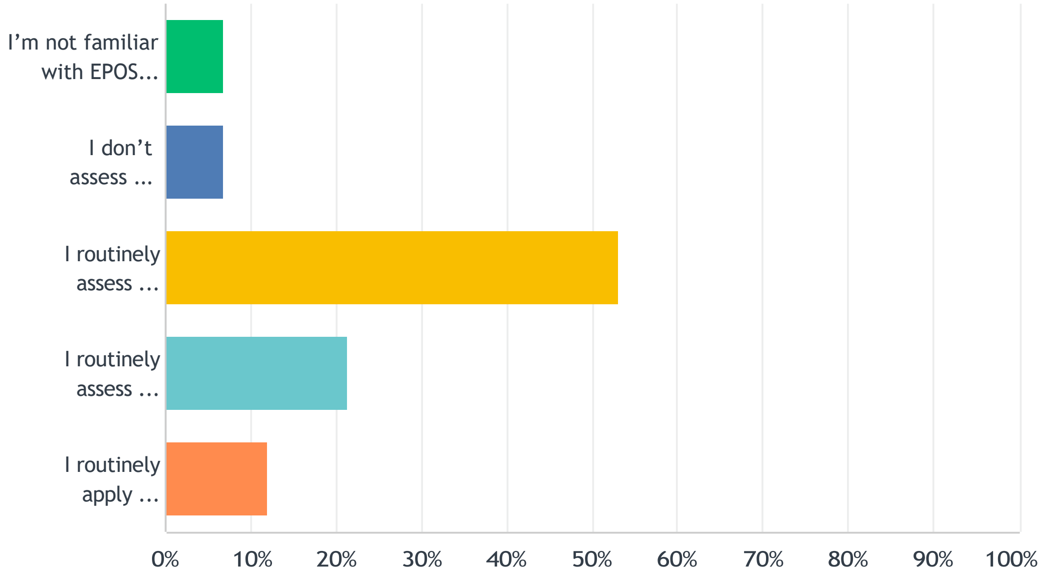

RESPONSE OPTIONS	RESPONSES	
I am not familiar with the EPOS 2020 guidelines	6.84%	8
I do not assess disease control according to the EPOS 2020 guidelines in clinical practice	6.84%	8
I routinely assess disease control as suggested by EPOS 2020 in all patients with CRSwNP	52.99%	62
I routinely assess disease control as suggested by EPOS 2020 in difficult to treat cases	21.37%	25
I routinely apply disease control assessment in candidates for a biological treatment	11.97%	14
TOTAL RESPONDERS		117

D12 Do you apply the indications of EUFOREA in clinical practice to define severe uncontrolled disease?

Responses: 117 Skipped: 0

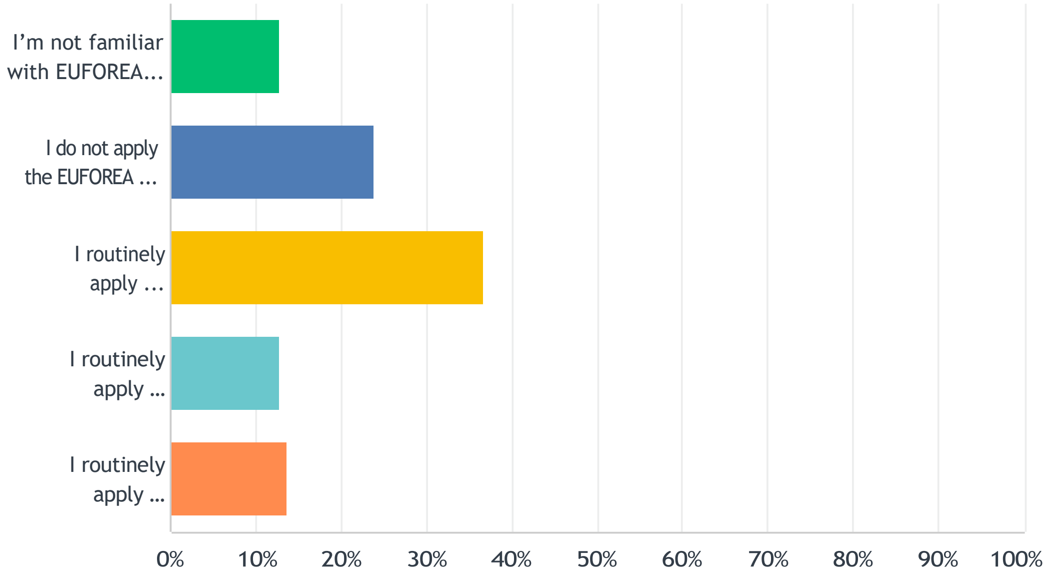

RESPONSE OPTIONS	RESPONSES	
I am not familiar with the EUFOREA guidelines	12.82%	15
I do not apply the indications of EUFOREA to define severe uncontrolled disease in patients with CRSwNP	23.93%	28
I routinely apply the indications of EUFOREA to define severe uncontrolled disease in patients with CRSwNP	36.75%	43
I routinely apply the indications of EUFOREA to define severe uncontrolled disease in difficult to treat cases	12.82%	15
I routinely apply the indications of EUFOREA to define severe uncontrolled disease in candidates for a biological treatment	13.68%	16
TOTAL RESPONDERS		117

D13 In which aspects are GUIDELINES lacking considering the definition of non-control in CRSwNP? (Multiple answers possible)

Responses: 117 Skipped: 0

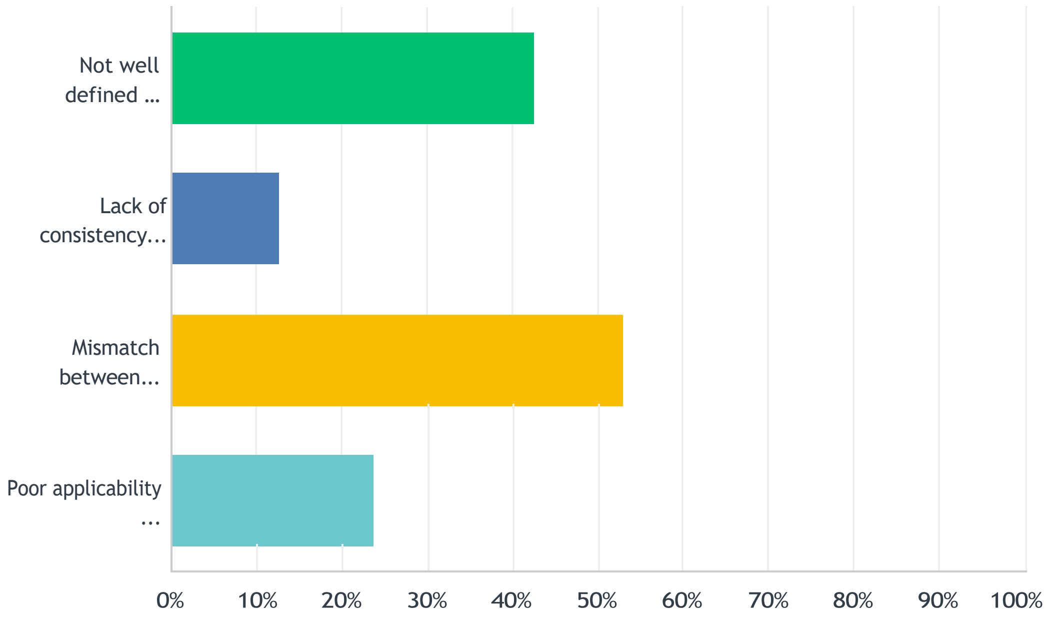

RESPONSE OPTIONS	RESPONSES	
The characteristics of the uncontrolled patients are not well defined	42.74%	50
Lack of consistency between different sources	12.82%	15
Mismatch between cut-off scores	52.99%	62
Poor applicability in clinical practice	23.93%	28
TOTAL RESPONDENTS		117

D14 Which of the following aspects can influence assessment of non-control in CRSwNP (multiple answers possible):

Responses: 117 Skipped: 0

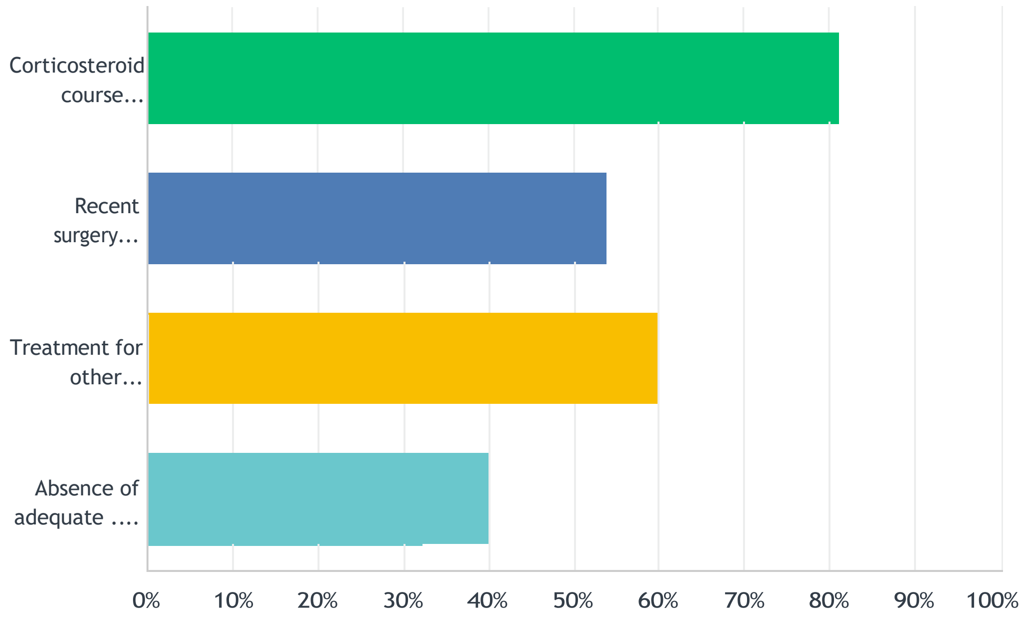

RESPONSE OPTIONS	RESPONSES	
Corticosteroid course close to or concurrent with evaluation	81.20%	95
Recent surgery	53.85%	63
Treatment for other comorbidities (related or unrelated)	59.83%	70
Absence of adequate diagnostic tools	32.48%	38
TOTAL RESPONDERS		117

D15 Which of these criteria do you consider important in assessing disease control in CRSwNP in your clinical practice? (Multiple answers possible)

Responses: 117 Skipped: 0

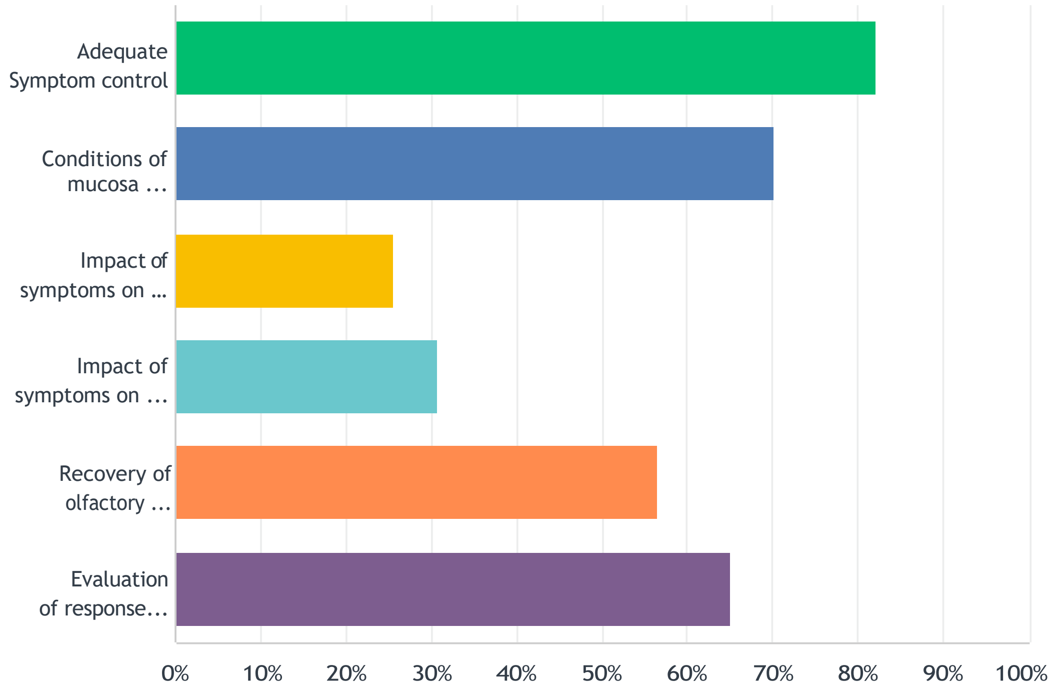

RESPONSE OPTIONS	RESPONSES	
Adequate symptom control	82.05%	96
Condition of mucosa assessed endoscopically (secretions and polypoid neoformations)	70.09%	82
Impact of symptoms on sleep	25.64%	30
Impact of symptoms on job performance	30.77%	36
Recovery of olfactory functionality	56.41%	66
Evaluation of response to previous treatments (corticosteroids, surgery o biological threapies	64.96%	76
TOTAL RESPONDERS		117

D16 In your clinical practice, which symptoms do you think are most important in defining non-control in CRSwNP? (Multiple answers possible)

Responses: 117 Skipped: 0

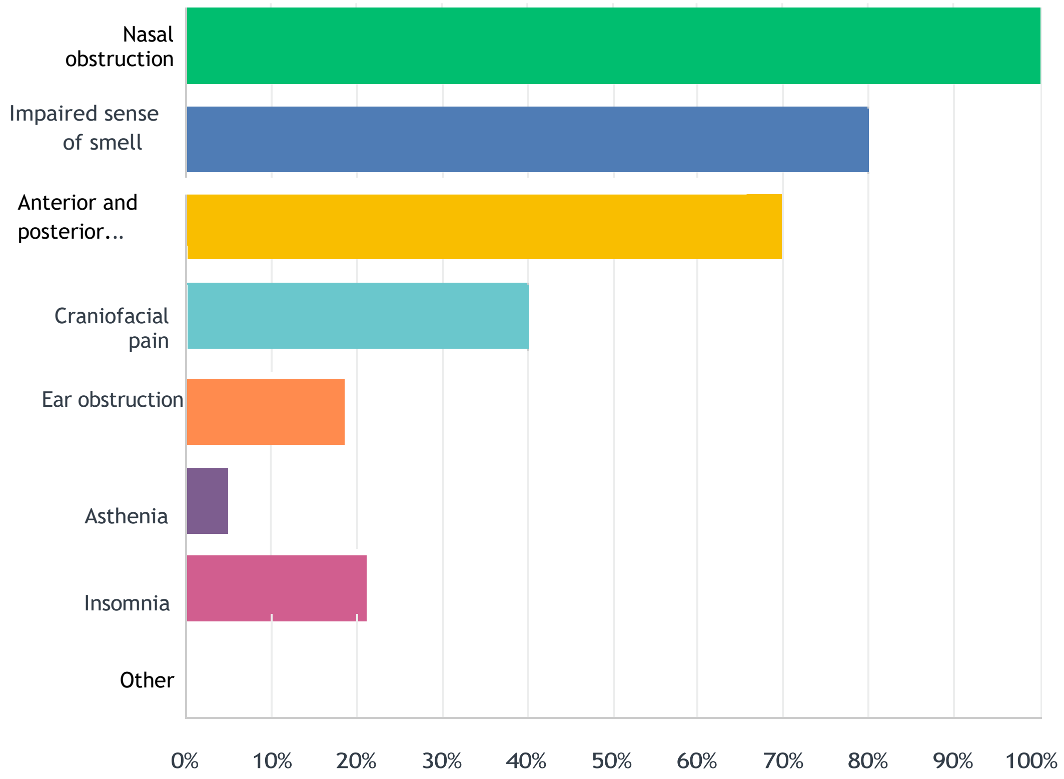

RESPONSE OPTIONS	RESPONSES	
Nasal obstruction	94.02%	110
Impaired sense of smell	80.34%	94
Anterior and posterior rhinorrhea	64.96%	76
Craniofacial pain	41.03%	48
Ear obstruction	18.80%	22
Asthenia	5.13%	6
Insomnia	21.37%	25
Other	0.00%	0
TOTAL RESPONDERS		117

D17 In your clinical practice, do you think it is important to have a well-defined score and a relative cut-off for each symptom that defines non-control in CRSwNP?

Responses: 117 Skipped: 0

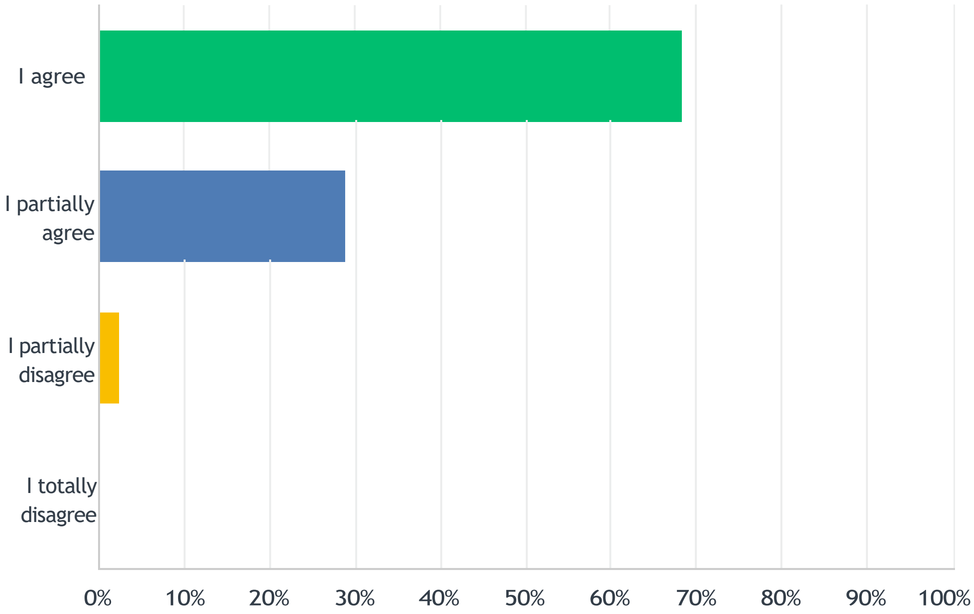

RESPONSE OPTIONS	RESPONSES	
I agree	68.38%	80
I partially agree	29.06%	34
I partially disagree	2.56%	3
I totally disagree	0.00%	0
TOTAL RESPONDERS		117

D18 To what extent do you agree that the main symptoms to consider are nasal obstruction, rhinorrhea and smell, to define an uncontrolled patient?

Responses: 117 Skipped: 0

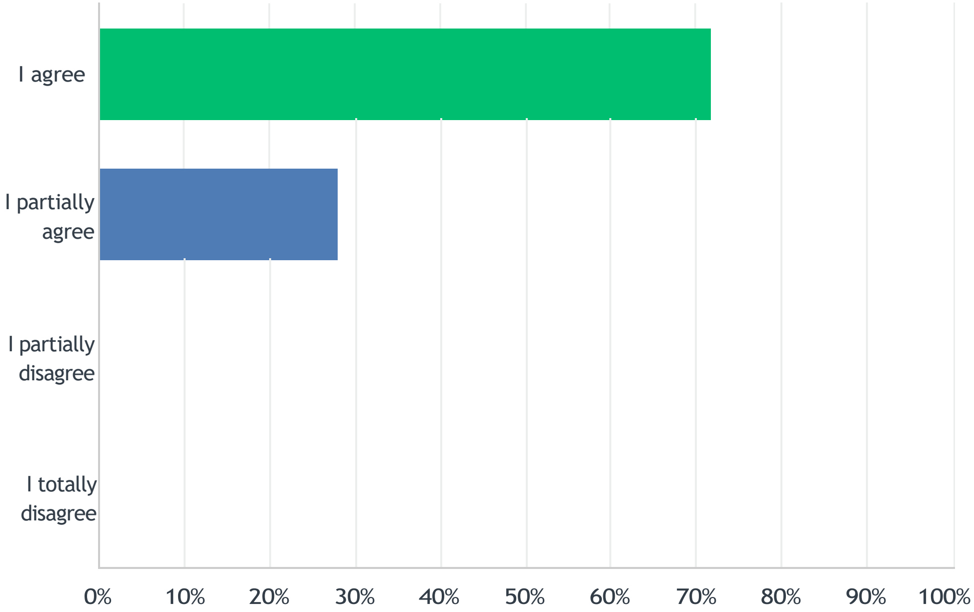

RESPONSE OPTIONS	RESPONSES	
I agree	71.79%	84
I partially agree	28.21%	33
I partially disagree	0.00%	0
I totally disagree	0.00%	0
TOTAL RESPONDERS		117

D19 In your clinical practice, which aspects of QoL do you consider most important to define non-control in severe CRSwNP in surgically-naive patients (multiple answers possible):

Responses: 117 Skipped: 0

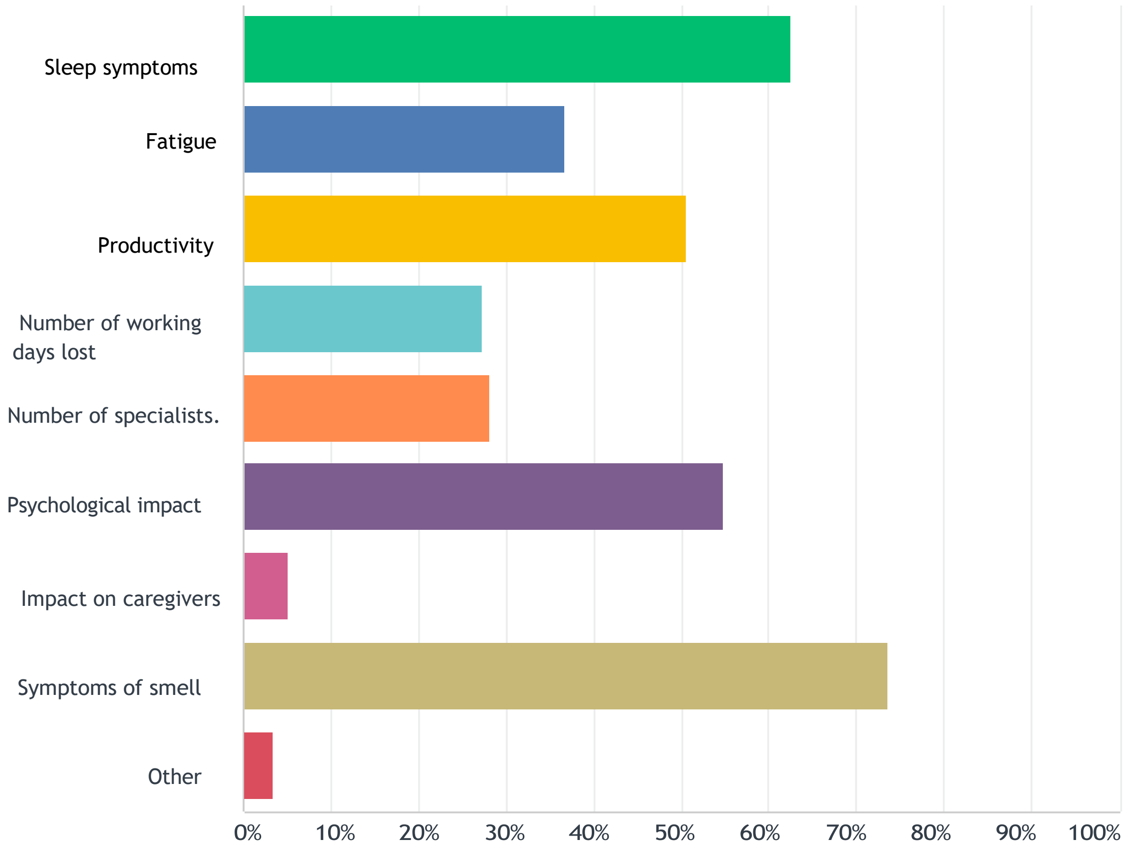

RESPONSE OPTIONS	RESPONSES	
Sleep symptoms	62.39%	73
Fatigue	36.75%	43
Productivity	50.43%	59
Number of working days lost	27.35%	32
Number of specialist visit per year	28.21%	33
Psychological impact	54.70%	64
Impact on caregivers	5.13%	6
Symptoms of smell	73.50%	86
Other	3.42%	4
TOTAL RESPONDERS		117

D20 Do you agree with the statement that the most important QoL aspects to consider in the specific evaluation of non-control are: asthenia, sleep quality and productivity at work? (Multiple answers possible)

Responses: 117 Skipped: 0

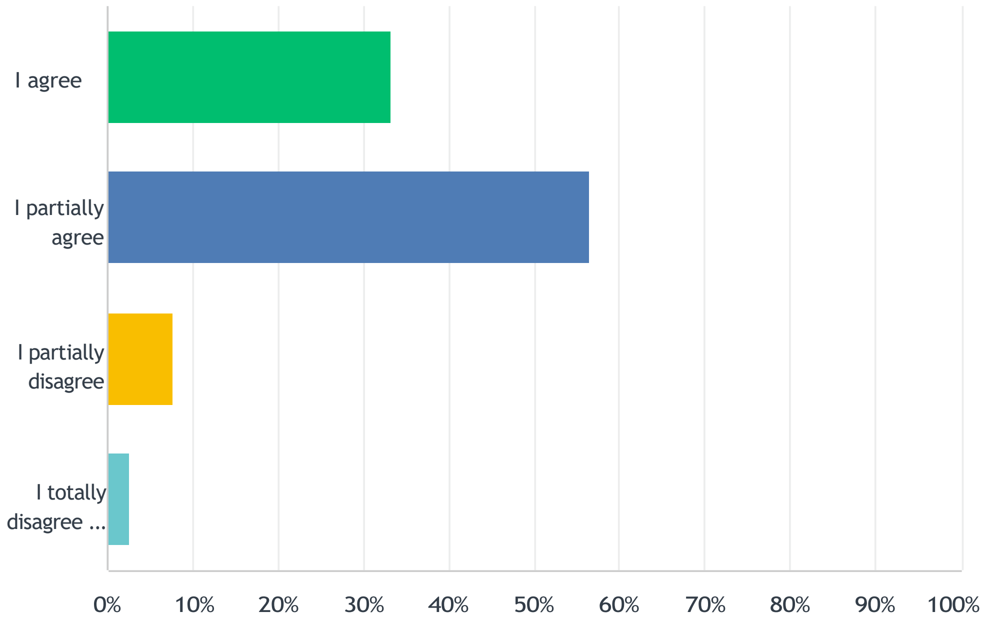

RESPONSE OPTIONS	RESPONSES	
I agree	33.33%	39
I partially agree	56.41%	66
I partially disagree	7.69%	9
I totally disagree	2.56%	3
TOTAL RESPONDERS		117

D21 Which of the following parameters do you think can help in defining a severe uncontrolled form? (multiple)

Responses: 117 Skipped: 0

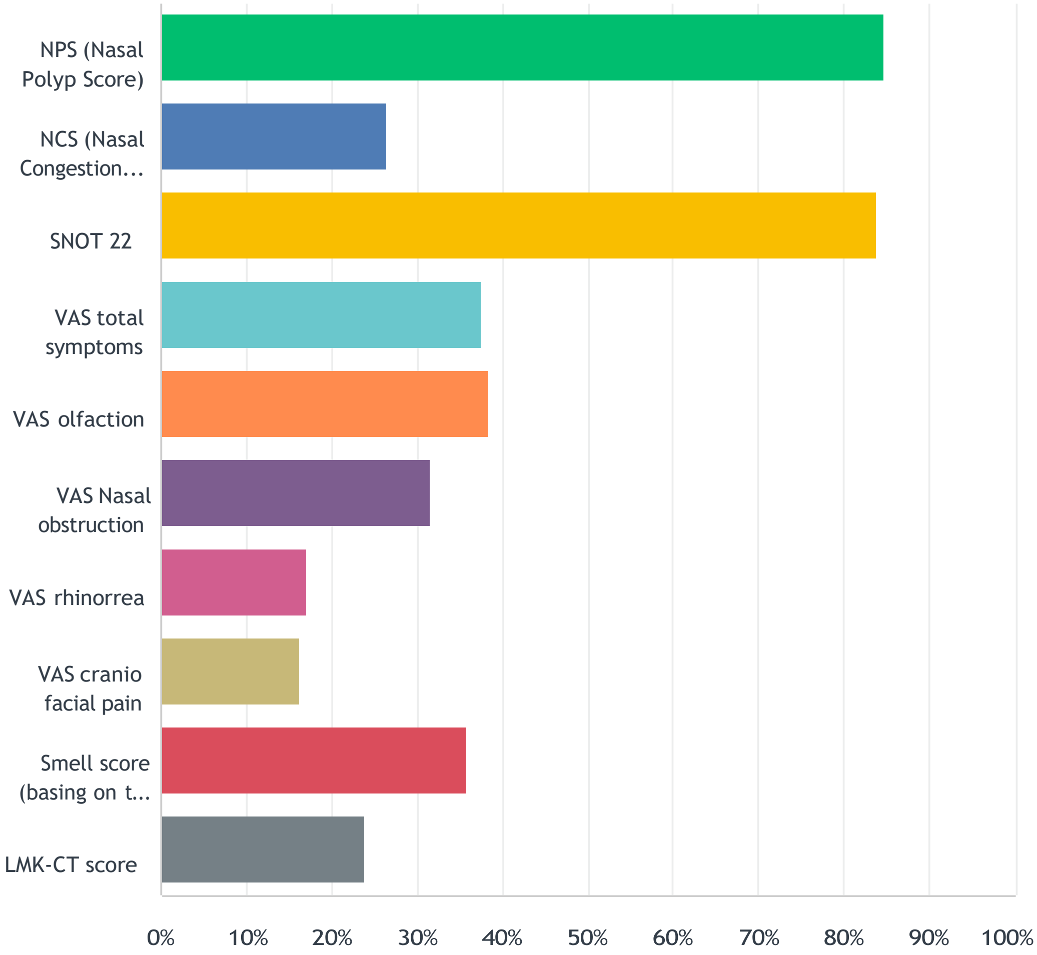

RESPONSE OPTIONS	RESPONSES	
NPS (Nasal Polyp Score)	84.62%	99
NCS (Nasal Congestion Score)	26.50%	31
SNOT 22	83.76%	98
VAS total symptoms	37.61%	44
VAS olfaction	38.46%	45
VAS Nasal obstruction	31.62%	37
VAS rhinorrea	17.09%	20
VAS cranio facial pain	16.24%	19
Smell score (basing on the test used)	35.90%	42
LMK-CT score	23.93%	28
TOTAL RESPONDENTS		117

D22 Regarding NPS, which of these values do you think is the most suitable to define a severe uncontrolled form?

Responses: 117 Skipped: 0

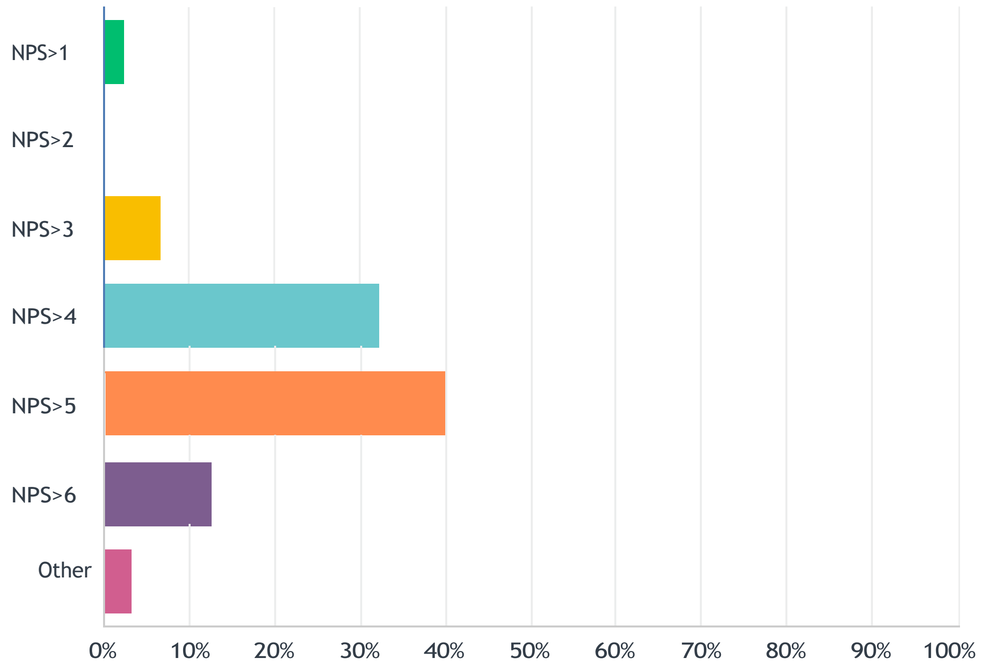

RESPONSE OPTIONS	RESPONSES	
NPS>1	2.56%	3
NPS>2	1.71%	2
NPS>3	6.84%	8
NPS>4	32.48%	38
NPS>5	40.17%	47
NPS>6	12.82%	15
Other	3.42%	4
TOTAL RESPONDERS		117

D23 Regarding SNOT-22 which of these values do you think is more suitable to define a severe uncontrolled form?

Responses: 117 Skipped: 0

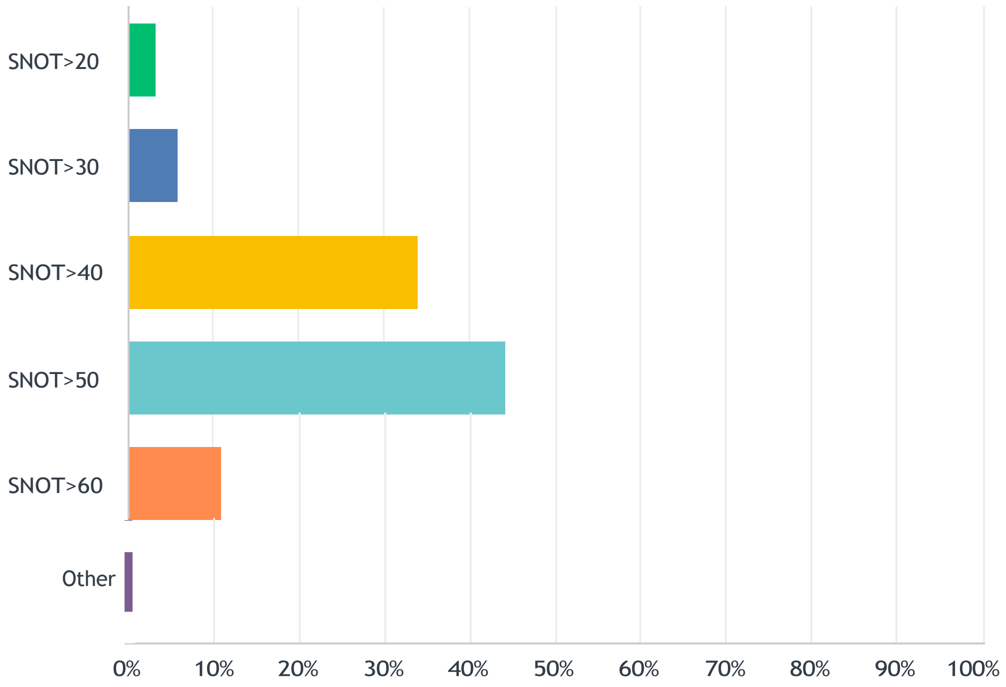

RESPONSE OPTIONS	RESPONSES	
SNOT>20	3.42%	4
SNOT>30	5.98%	7
SNOT>40	34.19%	40
SNOT>50	44.44%	52
SNOT>60	11.11%	13
Other	0.85%	1
TOTAL RESPONDERS		117

D24 In assessing QoL with SNOT-22, non-specific sinus domains should be evaluated since they are closely related to disease

Responses: 117 Skipped: 0

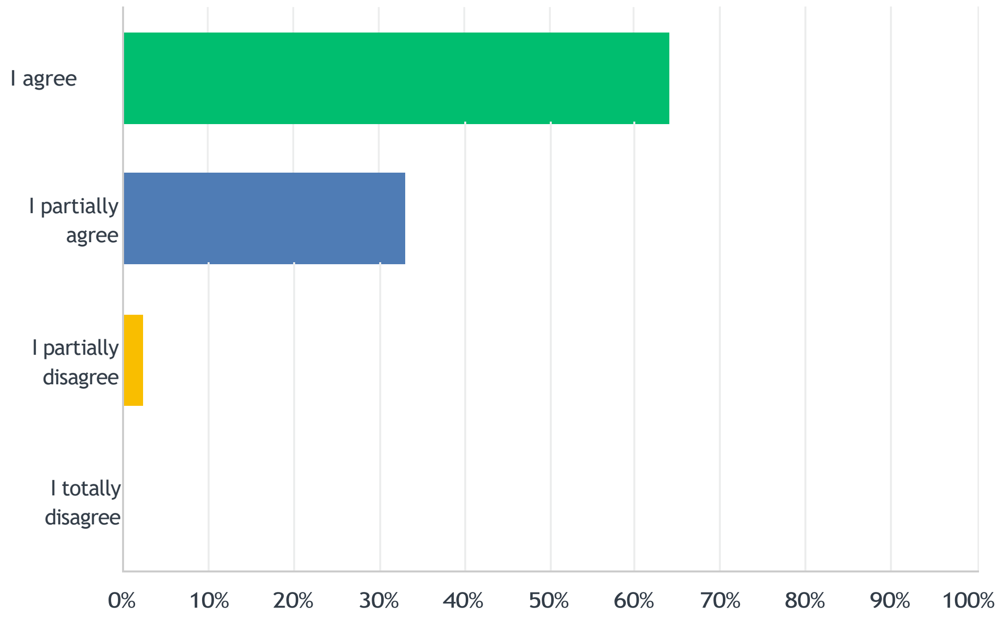

RESPONSE OPTIONS	RESPONSES	
I agree	64.10%	75
I partially agree	33.33%	39
I partially disagree	2.56%	3
I totally disagree	0.00%	0
TOTAL RESPONDERS		117

D25 With regard to the NCS, which of these values do you think is most suitable for defining a severe, uncontrolled form?

Responses: 117 Skipped: 0

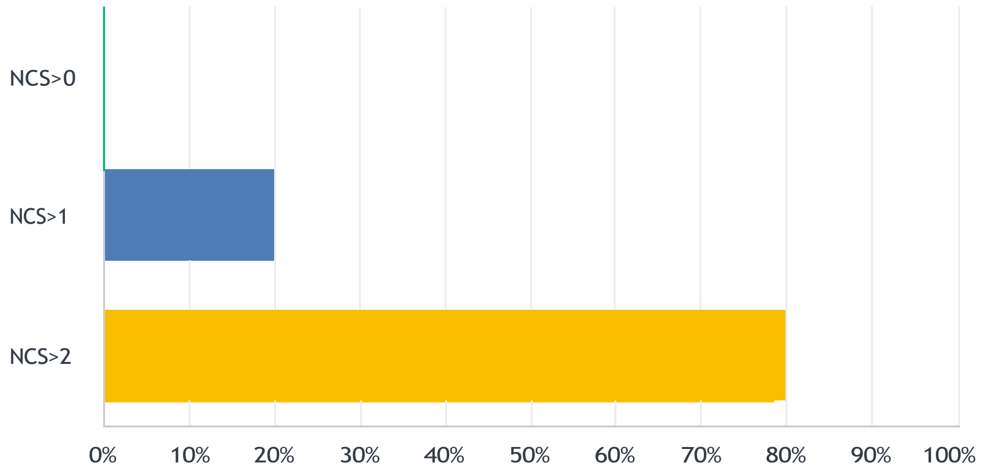

RESPONSE OPTIONS	RESPONSES	
NCS>0	1.71%	2
NCS>1	19.66%	23
NCS>2	78.63%	92
TOTAL RESPONDERS		117

D26 With regard to VAS total symptoms or specific for symptoms, which of these values do you think is more suitable to define a severe uncontrolled form?

Responses: 117 Skipped: 0

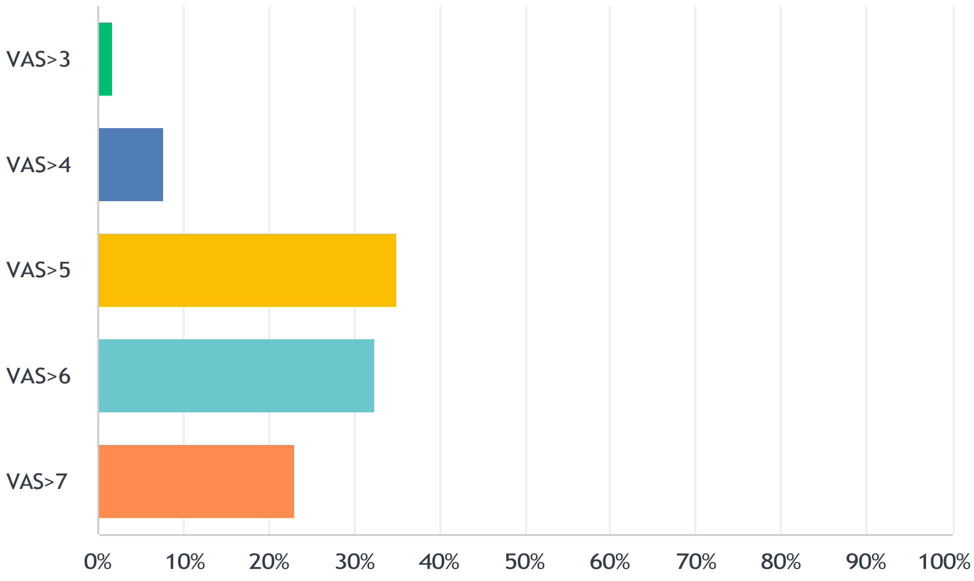

RESPONSE OPTIONS	RESPONSES	
VAS>3	1.71%	2
VAS>4	7.69%	9
VAS>5	35.04%	41
VAS>6	32.48%	38
VAS>7	23.08%	27
TOTAL RESPONDERS		117

D27 What do you think is the best way to assess olfaction during specific assessment for disease control? (multiple answers possible)

Responses: 117 Skipped: 0

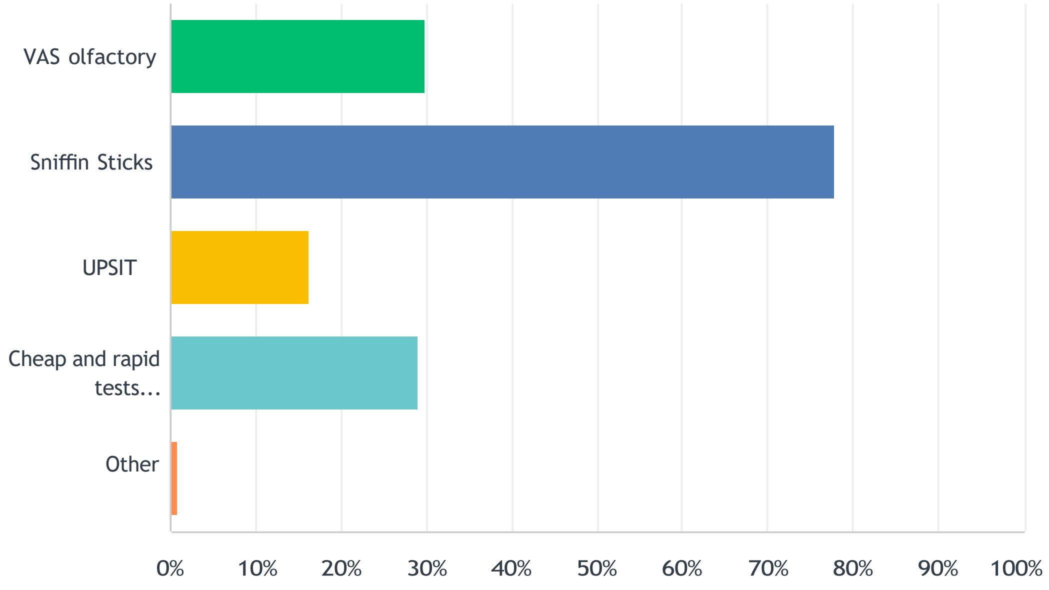

RESPONSE OPTIONS	RESPONSES	
VAS olfactory	29.91%	35
Sniffin Sticks	77.78%	91
UPSIT	16.24%	19
Cheap and rapid semi-objective tests are needed	29.06%	34
Other	0.85%	1
TOTAL RESPONDERS		117

D28 Considering previous surgeries, what do you think it is necessary to assess? (multiple answers possible)

Responses: 117 Skipped: 0

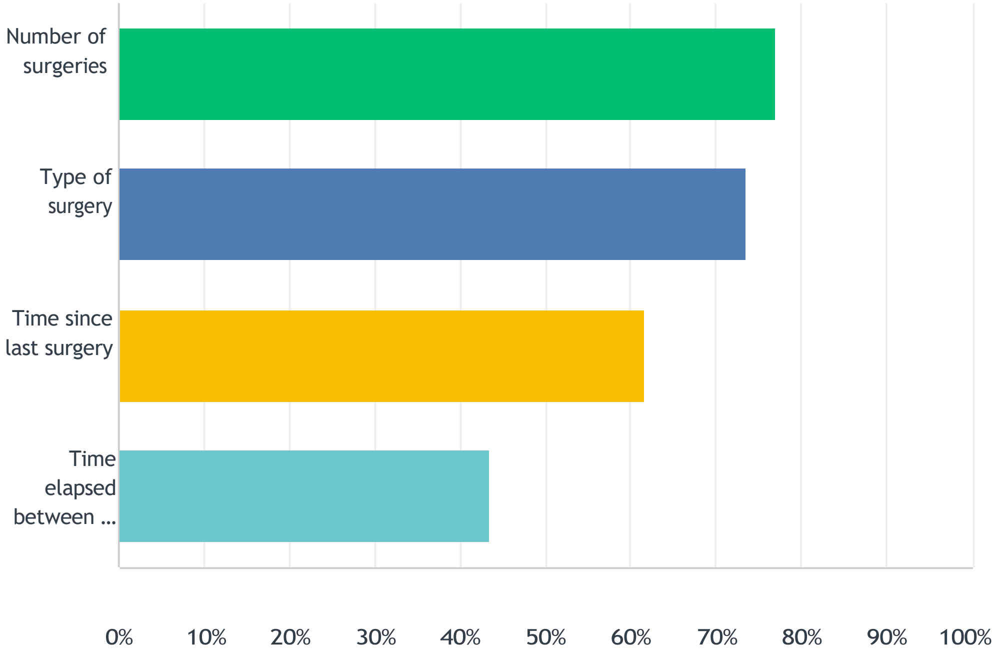

RESPONSE OPTIONS	RESPONSES	
Number of surgeries	76.92%	90
Type of surgery	73.50%	86
Time since last surgery	61.54%	72
Time elapsed between different surgeries	43.59%	51
TOTAL RESPONDENTS		117

D29 What percentage of CRSwNP patients are uncontrolled after surgery in your practice?

Responses: 117 Skipped: 0

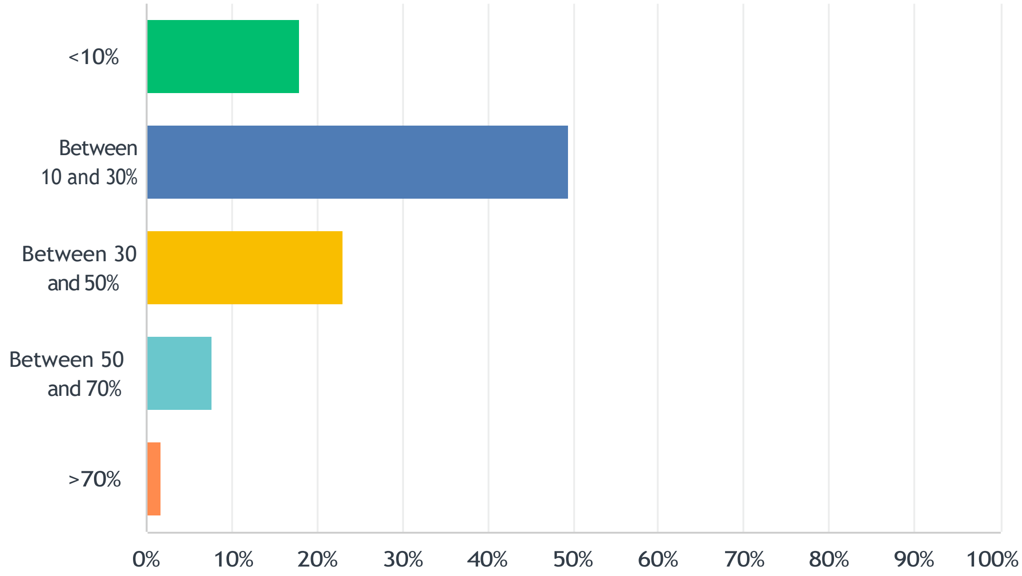

RESPONSE OPTIONS	RESPONSES	
<10%	17.95%	21
Between 10 and 30%	49.57%	58
Between 30 and 50%	23.08%	27
Between 50 and 70%	7.69%	9
>70%	1.71%	2
TOTAL RESPONDERS		117

D30 In your clinical practice, how long after the last intervention can it be held that surgery does not guarantee patient control?

Responses: 117 Skipped: 0

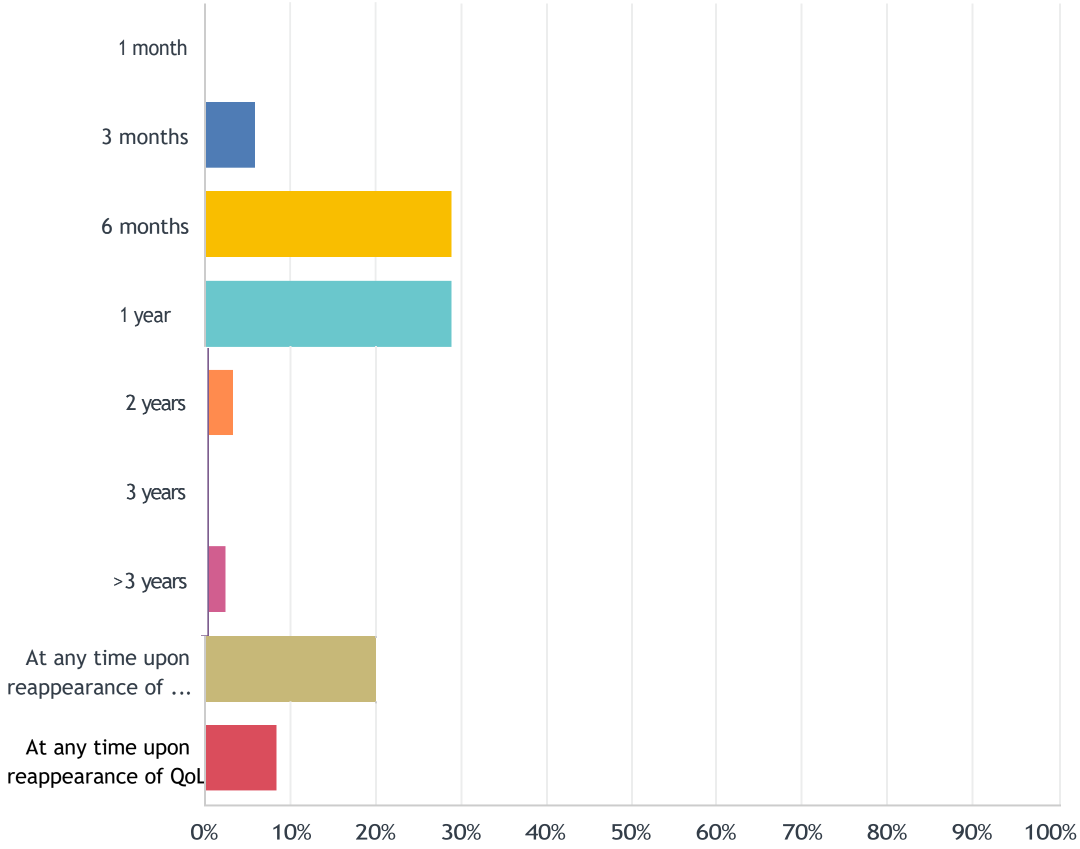

RESPONSE OPTIONS	RESPONSES	
1 month	0.00%	0
3 months	5.98%	7
6 months	29.06%	34
1 year	29.06%	34
2 years	3.42%	4
3 years	0.85%	1
>3 years	2.56%	3
At any time upon reappearance of objectivity on endoscopy (NPS)	20.51%	24
At any time upon reappearance of QoL symptoms (SNOT 22)	8.55%	10
TOTAL RESPONDERS		117

D31 To what extent do you agree that a patient, to be defined as uncontrolled, must have received adequate surgical treatment?

Responses: 117 Skipped: 0

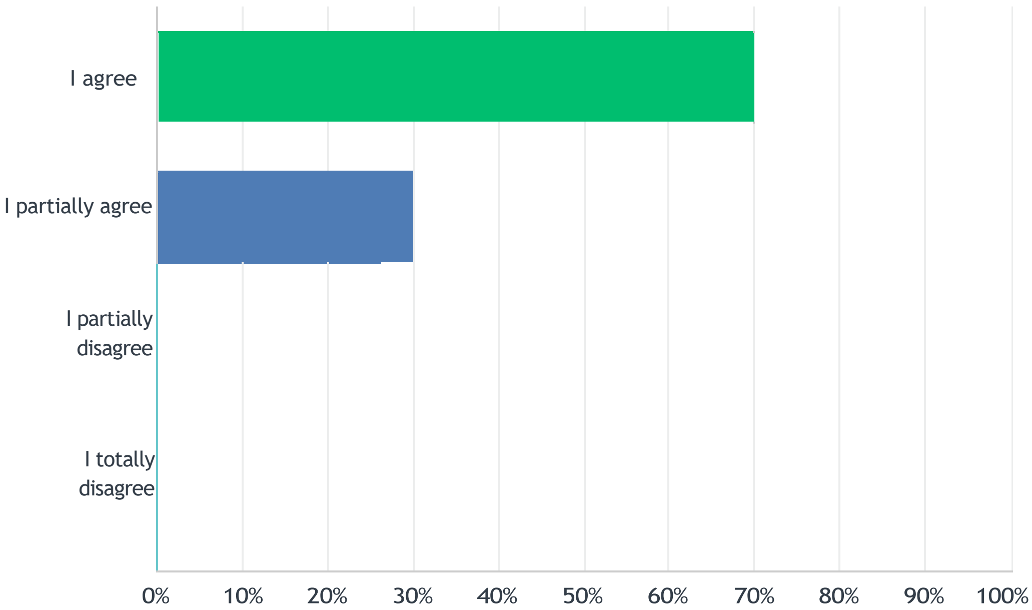

RESPONSE OPTIONS	RESPONSES	
I agree	70.09%	82
I partially agree	26.50%	31
I partially disagree	1.71%	2
I totally disagree	1.71%	2
TOTAL RESPONDERS		117

D32 In your clinical practice, how do you define adequate surgical therapy?

Responses: 117 Skipped: 0

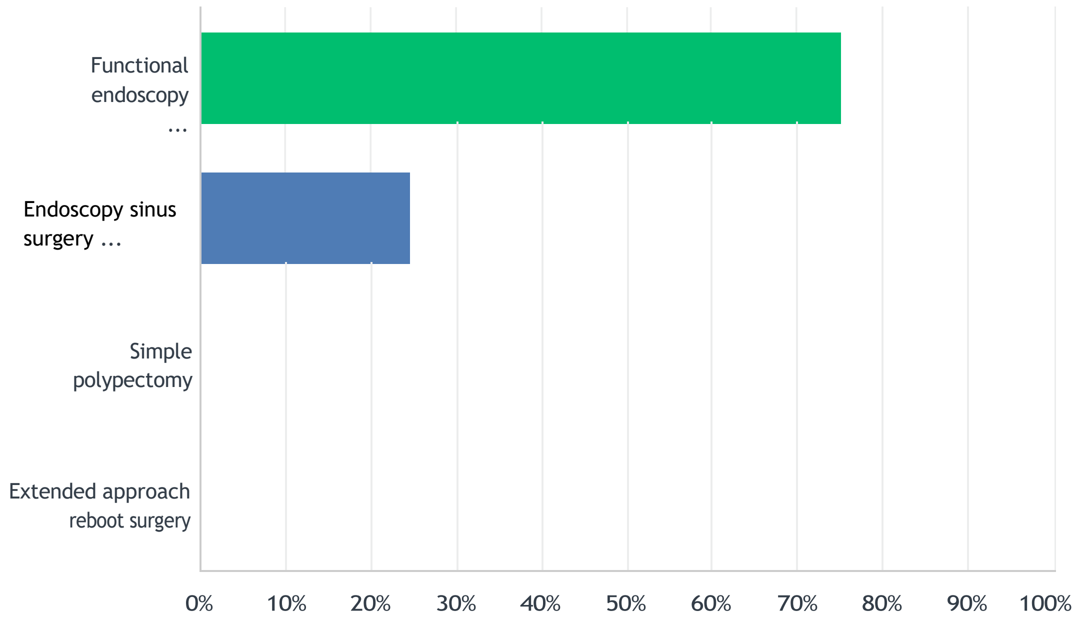

RESPONSE OPTIONS	RESPONSES	
Functional endoscopic sinus surgery with preservation of the middle turbinate (FESS)	75.21%	88
Endoscopic sinus surgery with removal of the middle turbinate (ESS)	24.79%	29
Simple polypectomy	0.00%	0
Extended approach reboot surgery	0.00%	0
TOTAL RESPONDERS		117

D33 In your clinical practice, after how many operations can it be held that surgery does not ensure patient control?

Responses: 117 Skipped: 0

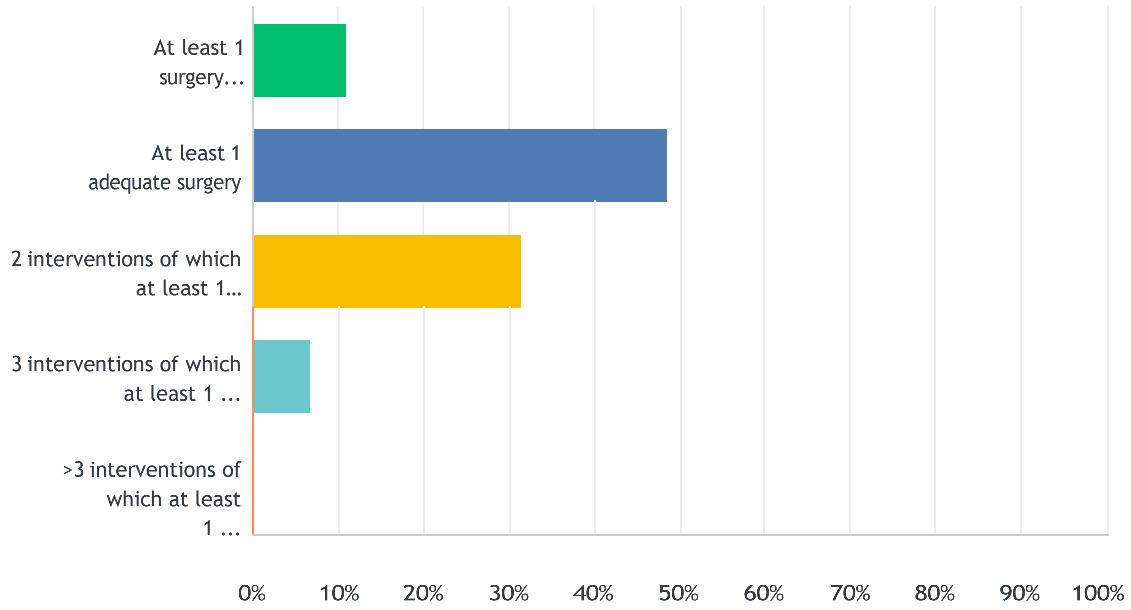

RESPONSE OPTIONS	RESPONSES	
At least 1 surgery	11.11%	13
At least 1 adequate surgery	48.72%	57
2 interventions of which at least 1 adequate	31.62%	37
3 interventions of which at least 1 adequate	6.84%	8
>3 interventions of which at least 1 adequate	1.71%	2
TOTAL RESPONDERS		117

D34 In a patient who has already undergone at least one surgery, which NPS score can determine a state of non-control?

Responses: 117 Skipped: 0

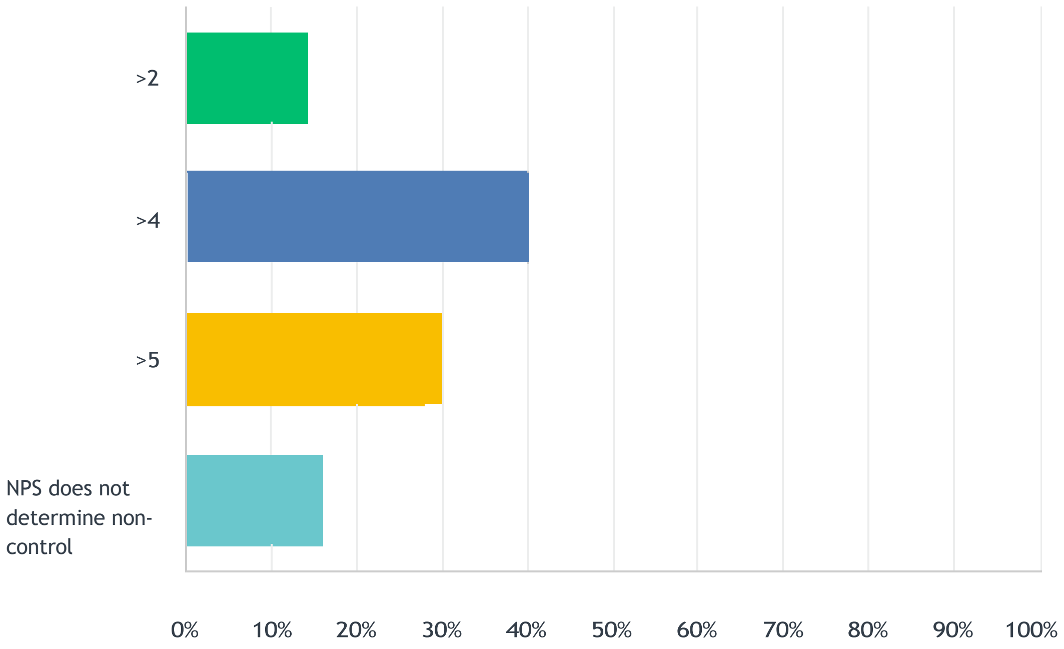

RESPONSE OPTIONS	RESPONSES	
>2	14.53%	17
>4	41.03%	48
>5	28.21%	33
NPS does not determine non-control	16.24%	19
TOTAL RESPONDERS		117

D35 In your clinical practice what aspects associated with OCS treatment do you use to define non-control in severe CRSwNP patient who is naïve to surgical treatment? (Multiple answers possible)

Responses: 117 Skipped: 0

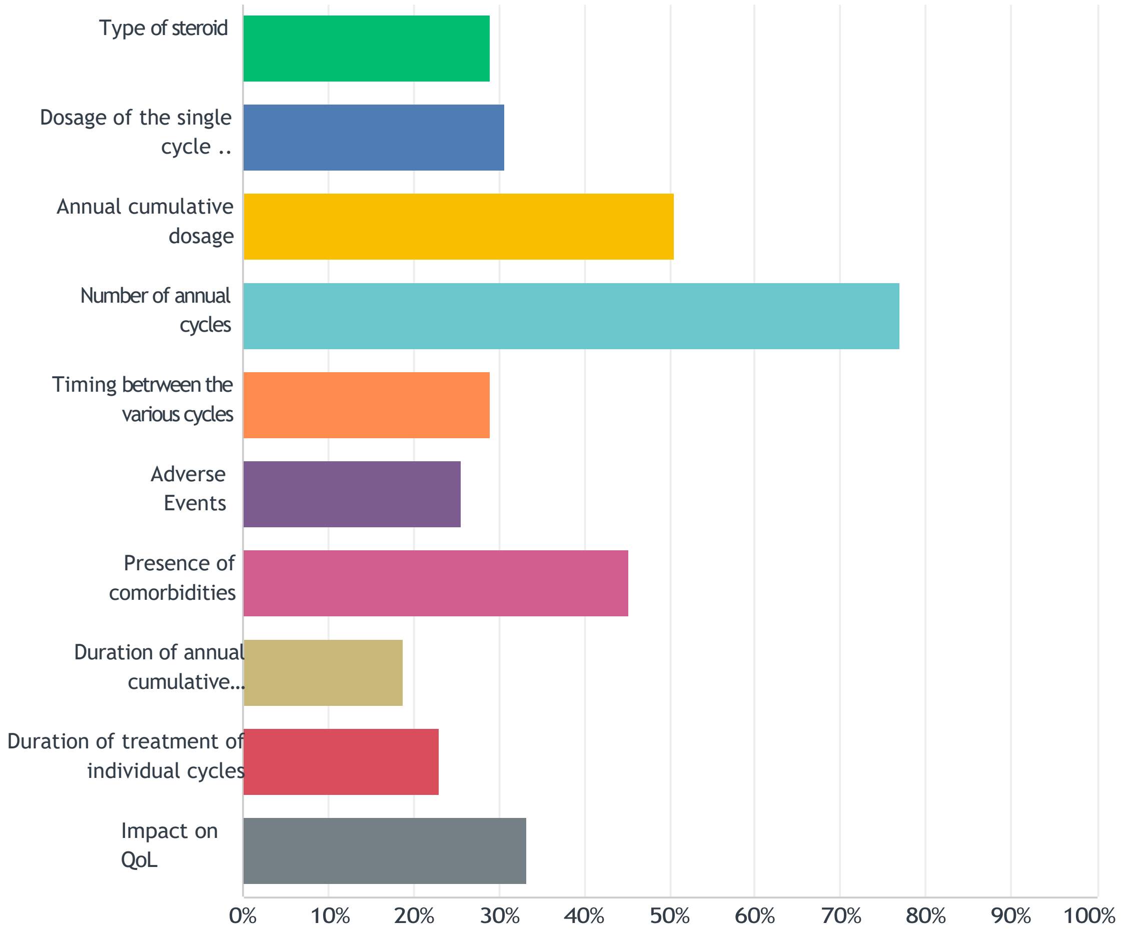

RESPONSE OPTIONS	RESPONSES	
Type of steroid	29.06%	34
Dosage of the single cycle	30.77%	36
Annual cumulative dosage	50.43%	59
Number of annual cycles	76.92%	90
Timing between the various cycles	29.06%	34
Adverse Events	25.64%	30
Presence of comorbidities	45.30%	53
Duration of annual cumulative treatment	18.80%	22
Duration of treatment of the individual cycles	23.08%	27
Impact on QoL	33.33%	39
TOTAL RESPONDENTS		117

D36 During disease control assessment when receiving INCS what do you think it is necessary… (multiple answers possible)

Responses: 117 Skipped: 0

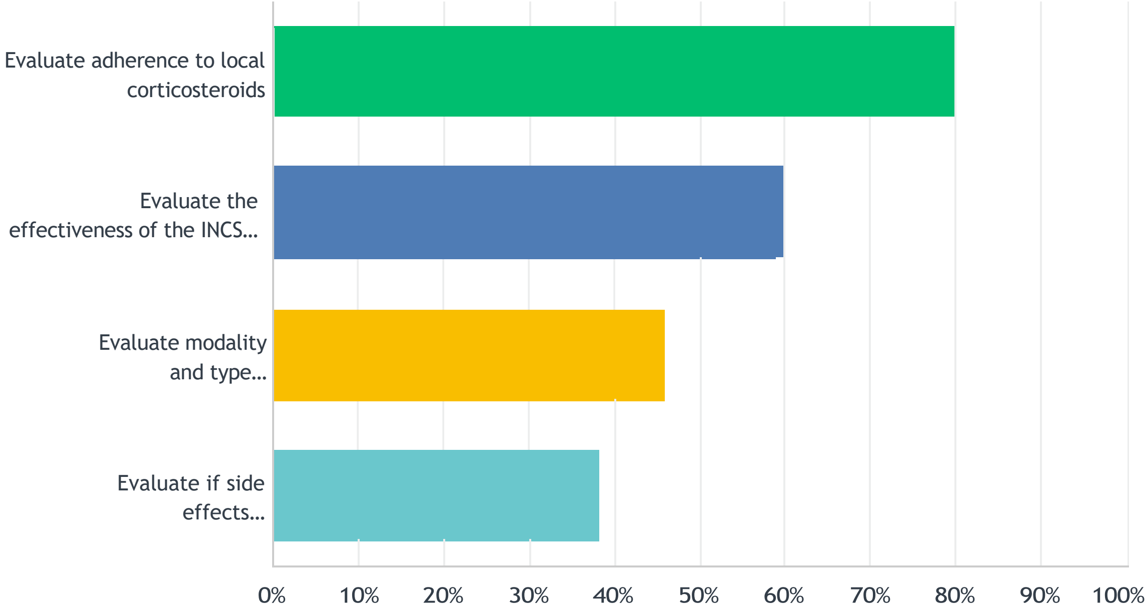

RESPONSE OPTIONS	RESPONSES	
Evaluate adherence to local corticosteroid	79.49%	93
Evaluate the effectiveness of the INCS after at least 2 months	58.97%	69
Evaluate modality and type of the corticosteroid taken	46.15%	54
Evaluate if side effects or contraindications have limited use	38.46%	45
TOTAL RESPONDENTS		117

D37 With regard to the evaluation of previous cortisone cycles per os, you consider the disease uncontrolled ...

Resposes: 117 Skipped: 0

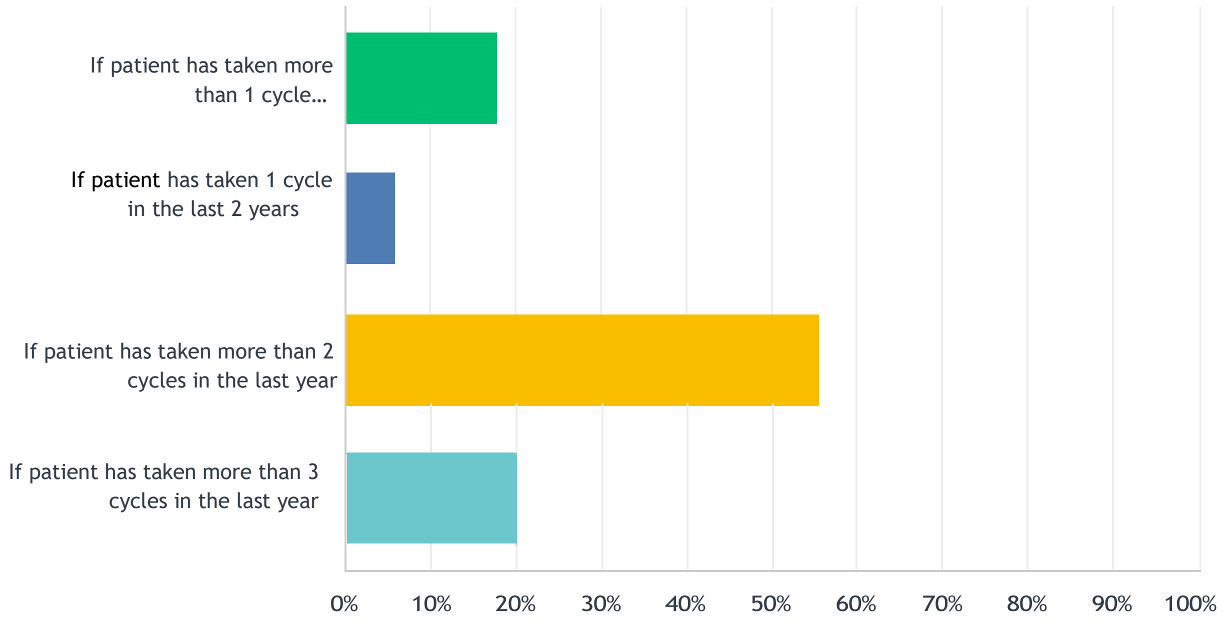

RESPONSE OPTIONS	RESPONSES	
If patient has taken more than 1 cycle in the last year	17.95%	21
If patient has taken 1 cycle in the last year two years	5.98%	7
If patient has taken more than 2 cycles in the last year	55.56%	65
If patient has taken more than 3 cycles in the last year	20.51%	24
TOTAL RESPONDENTS		117

D38 Do you agree that any complications related to the excessive use of corticosteroids (e.g. adrenal insufficiency, etc...) should be investigated in the evaluation of non-control?

Responses: 117 Skipped: 0

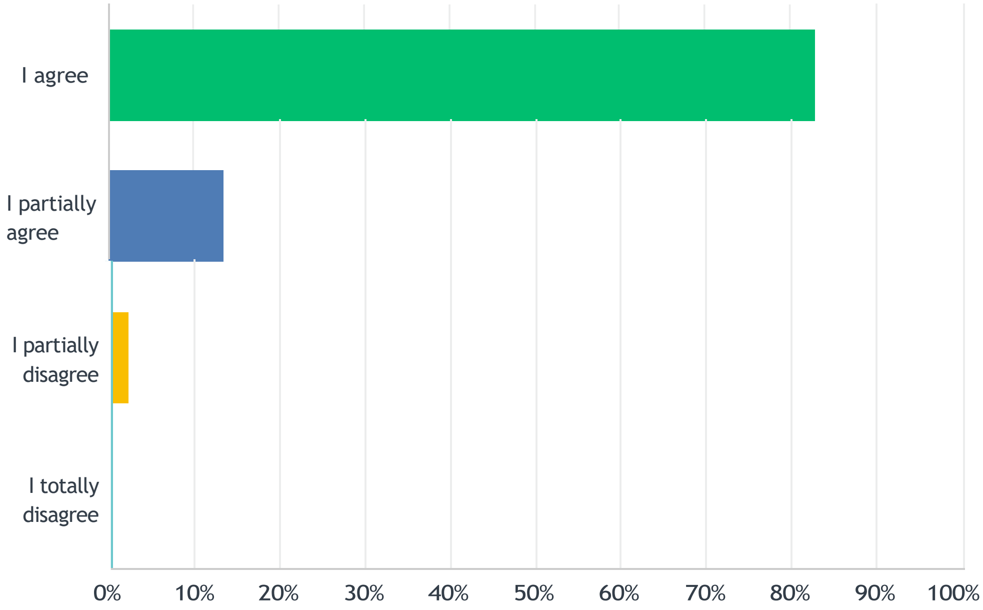

RESPONSE OPTIONS	RESPONSES	
I agree	82.91%	97
I partially agree	13.68%	16
I partially disagree	2.56%	3
I totally disagree	0.85%	1
TOTAL RESPONDERS		117

D39 In disease control evaluation of systemic corticosteroid, the following courses should be considered:

Responses: 117 Skipped: 0

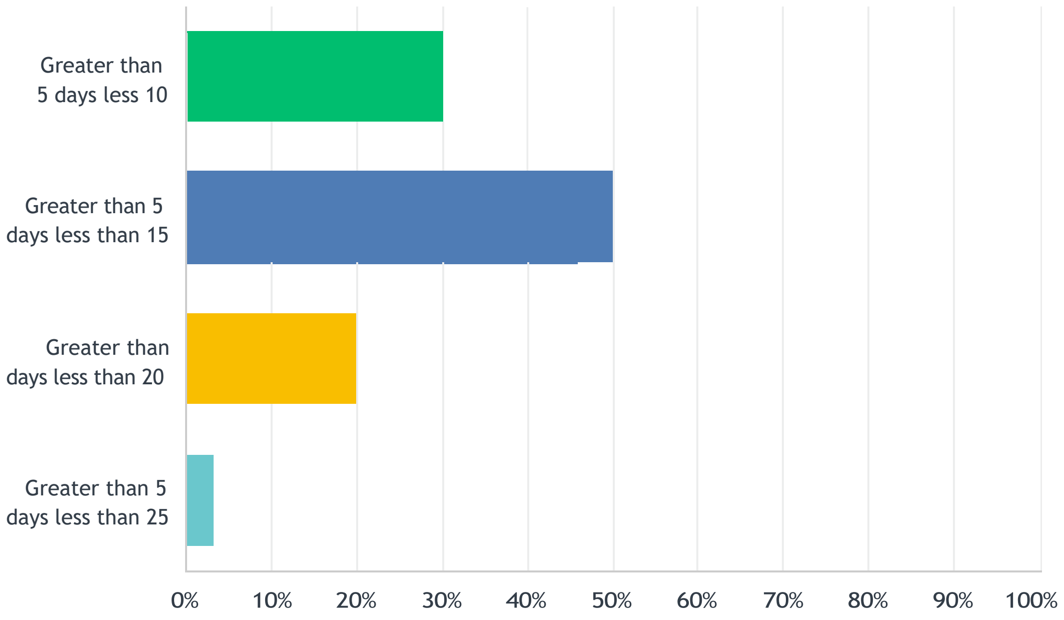

RESPONSE OPTIONS	RESPONSES	
Greater than 5 days less than 10	30.77%	36
Greater than 5 days less than 15	46.15%	54
Greater than 5 days less than 20	19.66%	23
Greater than 5 days less than 25	3.42%	4
TOTAL RESPONDERS		117

D40 Regarding previous biological treatments, do you think that the disease control (multiple answers possible):

Responses: 117 Skipped: 0

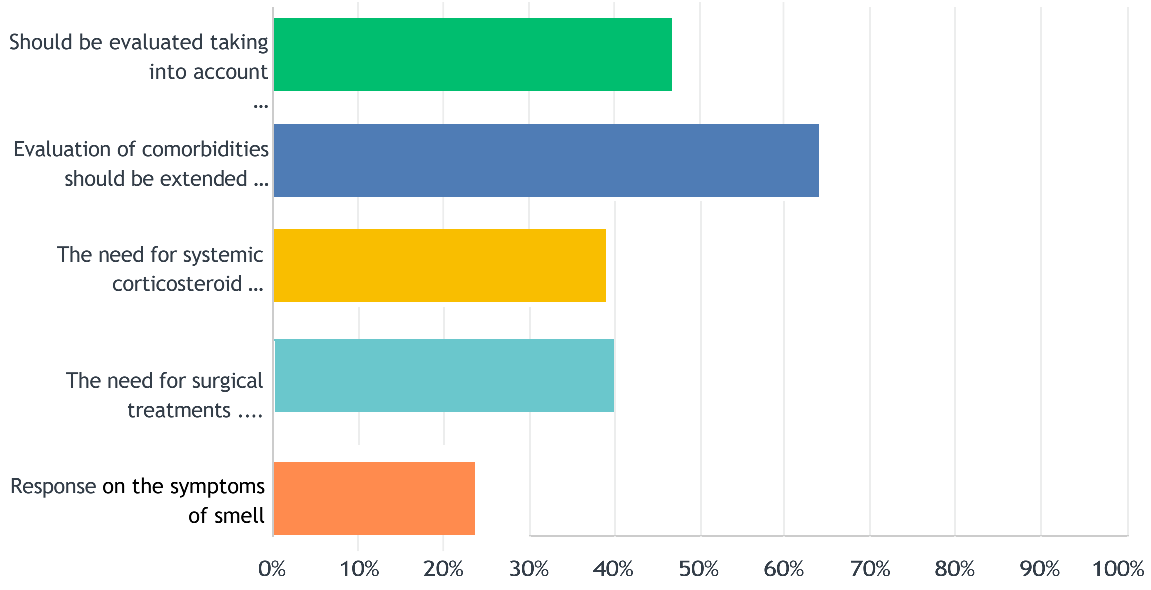

RESPONSE OPTIONS	RESPONSES	
Should be evaluated taking into consideration the same criteria listed above	47.01%	55
Evaluation of associated comorbidities should be extended to disease control	64.10%	75
The need for systemic corticosteroid therapy should be assessed during biologic therapy	39.32%	46
The need for surgical treatment should be assessed during biologic therapy	40.17%	47
It is necessary to evaluate response on the symptoms of smell	23.93%	28
TOTAL RESPONDERS		117

D41 Do you think it would be useful to have a reference grid as suggested by national disease control guidelines?

Responses: 117 Skipped: 0

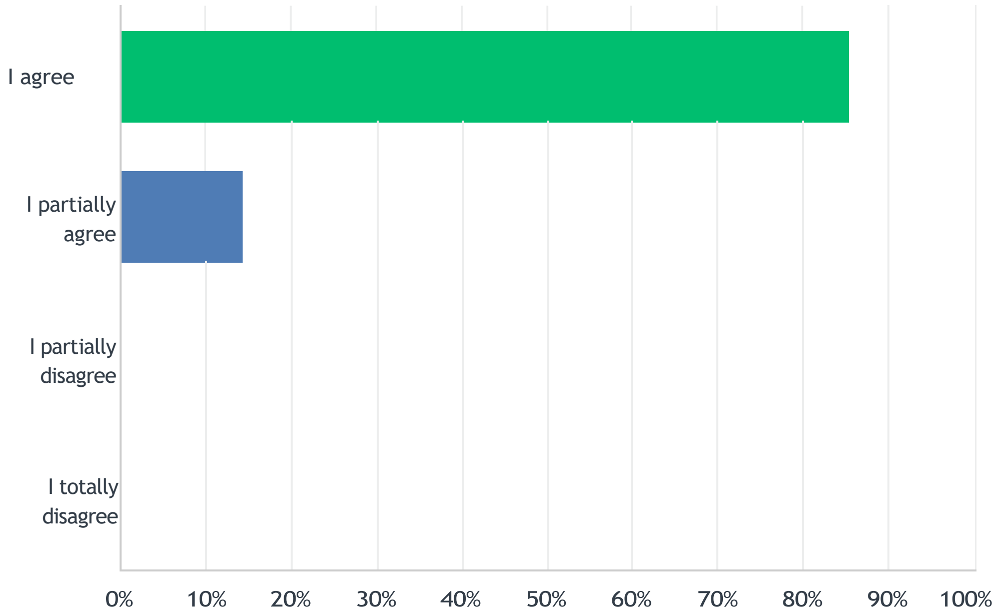

RESPONSE OPTIONS	RESPONSES	
I agree	85.47%	100
I partially agree	14.53%	17
I partially disagree	0.00%	0
I totally disagree	0.00%	0
TOTAL RESPONDERS		117

D42 In your clinical practice, what other pathologies do you consider as an additional element to define non-control in severe CRSwNP? (Multiple answers possible)

Responses: 117 Skipped: 0

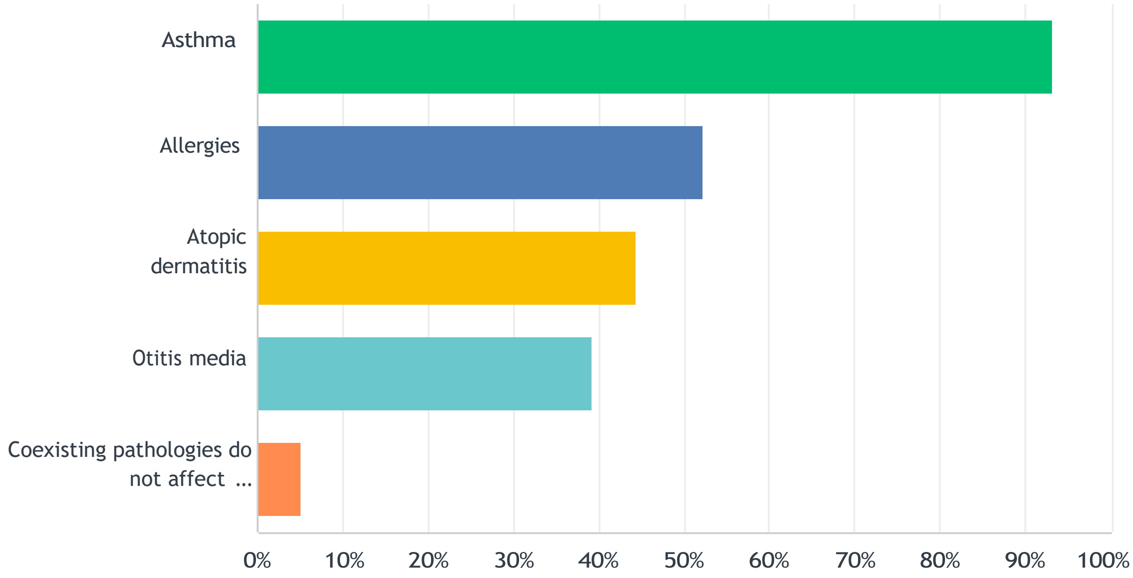

RESPONSE OPTIONS	RESPONSES	
Asthma	93.16%	109
Allergies	52.14%	61
Atopic dermatitis	44.44%	52
Otitis media	39.32%	46
Coexisting pathologies do not affect uncontrolled severe CRSwNP	5.13%	6
TOTAL RESPONSES		117

D43 In patients with asthma as a coexisting disease, asthma control also needs to be defined in your practice

Responses: 117 Skipped: 0

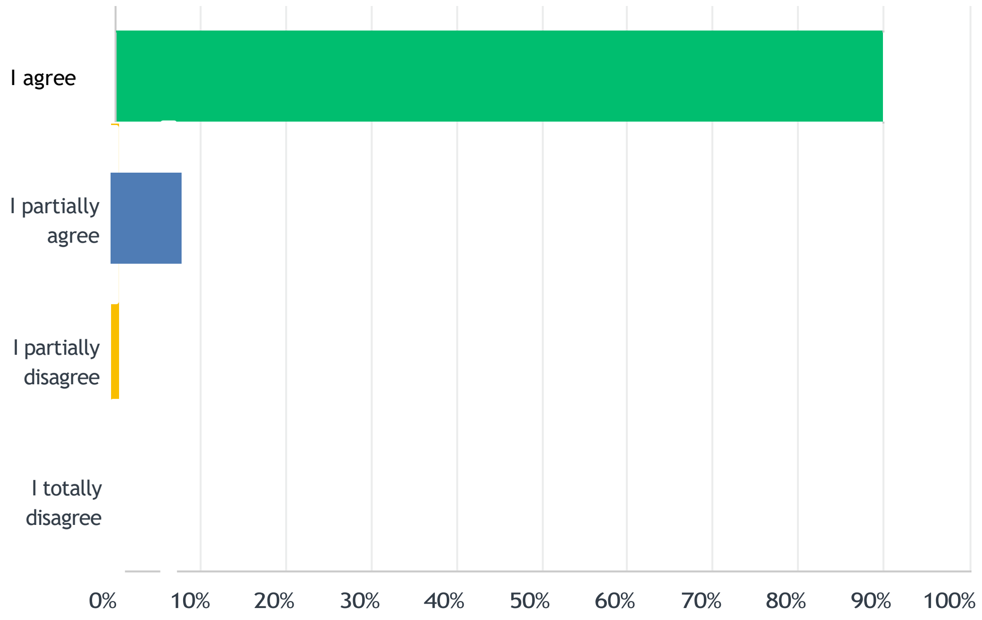

RESPONSE OPTIONS	RESPONSES	
I agree	90.60%	106
I partially agree	8.55%	10
I partially disagree	0.85%	1
I totally disagree	0.00%	0
TOTAL RESPONDERS		117

D44 Assessment by pulmonary specialists is important to assess asthma control

Responses: 117 Skipped: 0

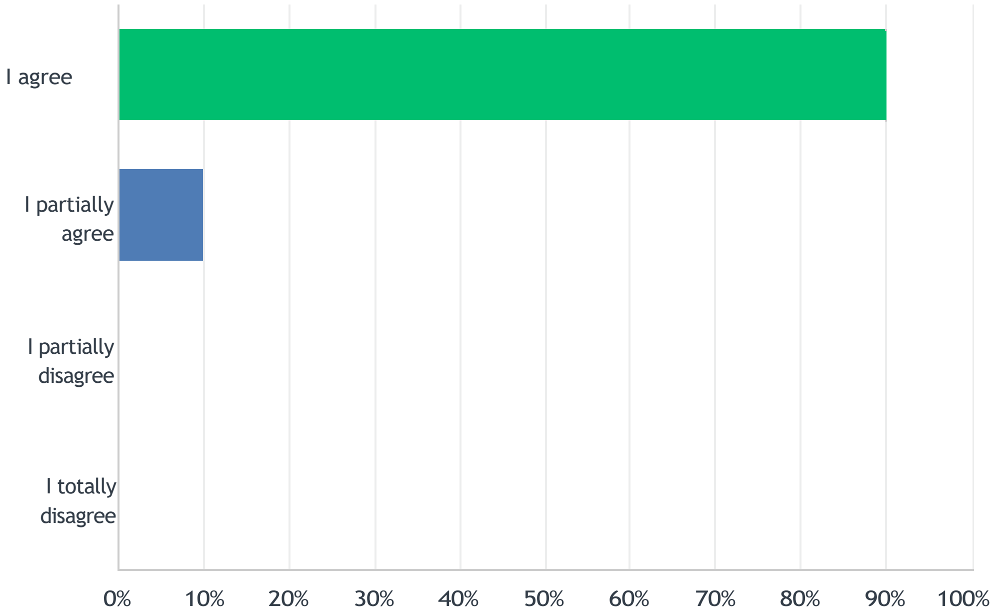

RESPONSE OPTIONS	RESPONSES	
I agree	90.60%	106
I partially agree	9.40%	11
I partially disagree	0.00%	0
I totally disagree	0.00%	0
TOTAL RESPONDERS		117

D45 Assessment of asthma and CRSwNP should be the subject of multidisciplinary discussion

Responses: 117 Skipped: 0

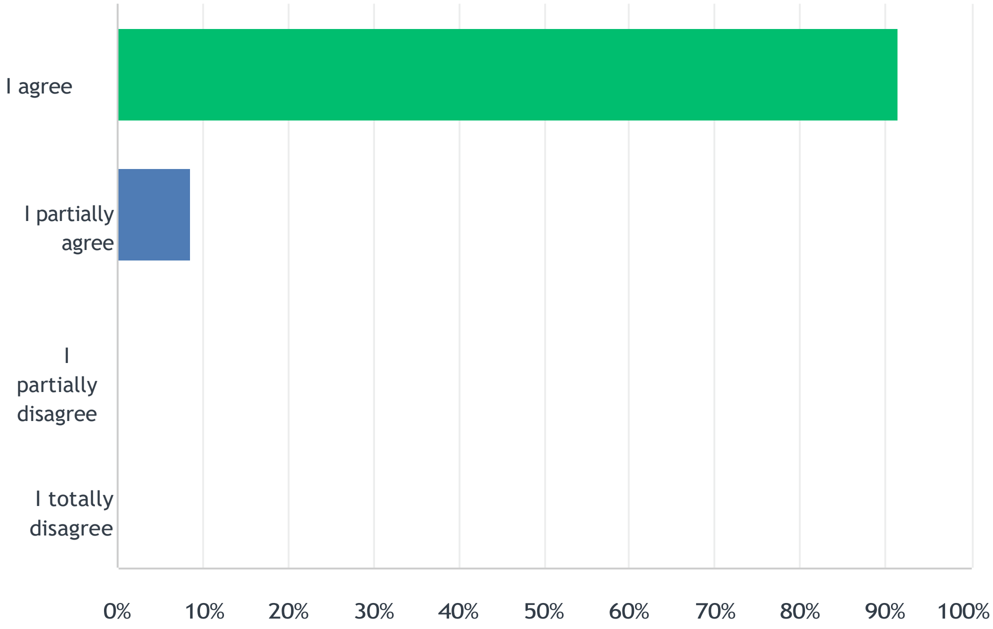

RESPONSE OPTIONS	RESPONSES	
I agree	91.45%	107
I partially agree	8.55%	10
I partially disagree	0.00%	0
I totally disagree	0.00%	0
TOTAL RESPONDERS		117

D46 It is important to have a therapeutic strategy that allows for control of both asthma and CRSwNP

Responses: 117 Skipped: 0

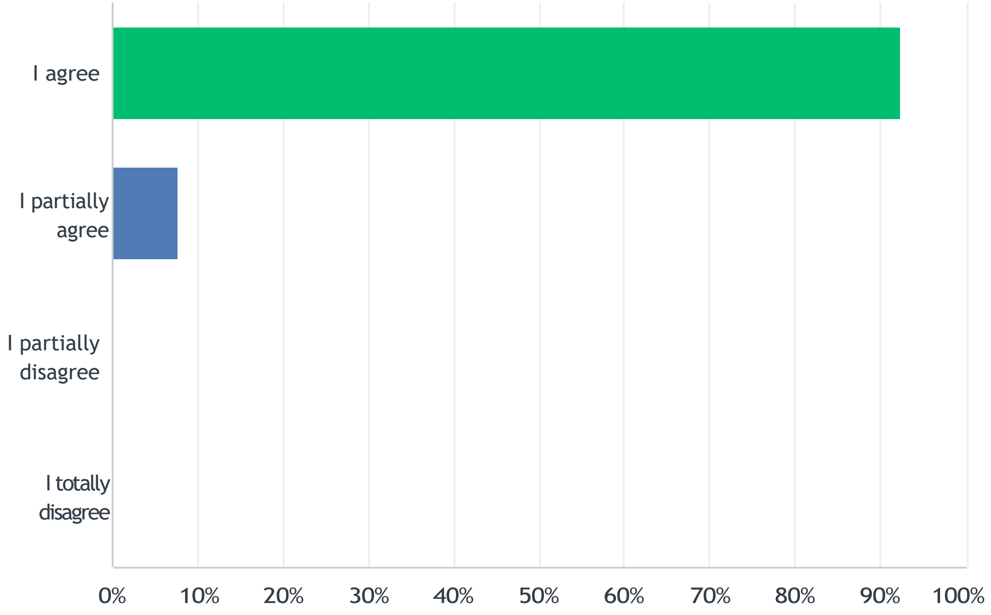

RESPONSE OPTIONS	RESPONSES	
I agree	92.31%	108
I partially agree	7.69%	9
I partially disagree	0.00%	0
I totally disagree	0.00%	0
TOTAL RESPONDERS		117
